# Sex hormone receptors, calcium-binding protein and Yap1 signaling regulate sex-dependent liver cell proliferation following partial hepatectomy

**DOI:** 10.1242/dmm.050900

**Published:** 2024-10-30

**Authors:** Mingkai Zhu, Yan Li, Qiaosen Shen, Zhiyuan Gong, Dong Liu

**Affiliations:** ^1^ School of Life Science, Southern University of Science and Technology, Shenzhen 518055, China; ^2^ Department of Biological Sciences, National University of Singapore, Singapore 117558

**Keywords:** Liver regeneration, Partial hepatectomy, Sex disparity, Zebrafish

## Abstract

Partial hepatectomy (PH) is commonly used to treat patients with hepatocellular carcinoma. The recovery of patients from PH depends on the initiation of liver regeneration, a process that mainly relies on liver cell proliferation. As sex affects the human liver regeneration progress, we investigated sex disparity in PH-induced liver regeneration in adult zebrafish. We found that, after PH, males began liver regeneration earlier than females in terms of liver cell proliferation and liver mass recovery, and this was associated with earlier activation of Yap1 signaling in male than female livers. We also found that androgen receptors regulated the sex-biased liver regeneration in a Yap1-dependent manner and that activated estrogen receptors are responsible for the later onset of female hepatocyte proliferation. Furthermore, we identified that S100A1, a calcium-binding protein, regulates the sex disparity in liver regeneration, as heterozygous S100A1 knockout inhibited Yap1 activity in male livers and delayed hepatocyte proliferation in males following PH. Thus, multiple pathways and/or their interplays contribute to the sex disparity in liver regeneration, suggesting that sex-biased therapeutic strategies are required for patients who have received PH-based therapies.

## Teaser

**Summary:** This study reveals the sex disparity in zebrafish liver cell proliferation following liver resection and how this disparity is regulated by sex hormone receptors and the calcium-binding protein S100A1.

## INTRODUCTION

Partial hepatectomy (PH) has been widely used in the clinic for treating hepatocellular carcinoma to improve patients' overall survival (Labgaa et al., 2020). As mature liver cells can re-enter the cell cycle upon liver injury, patients can regenerate their lost liver mass after a major PH (Michalopoulos, 2007). To reduce the risk of compromised liver regeneration and improve the outcome of PH-based therapies, a comprehensive understanding of PH-induced liver regeneration is of great importance.

Clinical reports have shown that male patients have a higher level of liver damage, whereas female patients have better recovery, after receiving PH (Birrer et al., 2022; Dong et al., 2022). Furthermore, sex disparity in the speed of liver regeneration has been demonstrated in rodent PH models despite conflicting results regarding which sex has earlier regeneration (Batmunkh et al., 2017; Wang et al., 2013; Zhou et al., 2016). This sex-biased liver regeneration has often been attributed to the responses of estrogen receptors (ERs) because PH can induce ER activation in the liver and estrogen promotes hepatocyte proliferation (Batmunkh et al., 2017; Francavilla et al., 1990; Kao et al., 2017; Uebi et al., 2015). Nevertheless, sex disparity in ER activity can only explain a female-biased liver regeneration but not a male-biased liver regeneration. Apart from sex hormone receptors, only a handful of factors such as miR-17∼92 and histone deacetylase have been suggested to be responsible for sex-biased liver regeneration (Wang et al., 2013; Zhou et al., 2016). Overall, although the existence of sex disparity in PH-induced liver regeneration has been widely reported, the mechanisms underlying this disparity have not been sufficiently explored.

The Hippo-YAP1 signaling pathway is known to regulate ectopic organ growth including organ regeneration and tumorigenesis (Hansen et al., 2015; Kowalczyk et al., 2022). In a rat PH model, YAP1 was activated shortly after the surgery and switched off gradually when reaching the full recovery of liver mass, suggesting its correlation with liver regeneration (Grijalva et al., 2014). Knockout (KO) of YAP1 has also been found to attenuate PH-induced liver regeneration in rats (Lu et al., 2018). The major upstream regulators of Hippo-YAP1 signaling are tyrosine receptor kinases (RTKs), but many other factors have been found to interact with this pathway (Hansen et al., 2015). Recently, S100A1, a calcium-binding protein from the S100 family, was suggested to promote YAP1 activity in human papillary thyroid carcinoma cells *in vitro* and *in vivo* (Wang et al., 2021). Although YAP1 activity has been demonstrated to drive liver regeneration following PH, its role in the sex disparity of liver regeneration remains unclear.

Zebrafish (*Danio rerio*) have been widely used for regeneration research because of their high regenerative capacity (Gao and Peng, 2021). To study PH-induced liver regeneration, the zebrafish PH model was developed over 15 years ago and has improved continuously (Kan et al., 2009; Oderberg and Goessling, 2021; Sadler et al., 2007). It has been employed to investigate the mechanism underlying PH-induced liver regeneration in several studies (Chen et al., 2020; Dovey et al., 2009; Goessling et al., 2009; Kan et al., 2009; Zhu et al., 2014). Kan et al. (2009) showed that male zebrafish recovered liver mass earlier than female zebrafish after receiving PH, which is consistent with the results from studies using the mouse PH model (Wang et al., 2013; Zhou et al., 2016). However, sex disparity in zebrafish liver regeneration has yet to be fully understood.

In this study, we confirmed sex disparity in zebrafish liver regeneration following PH and further explored the underlying mechanism(s). In general, males showed earlier liver cell proliferation and earlier recovery of liver mass than females did after receiving PH, and this earlier initiation was associated with earlier activation of the Yap1 signaling pathway and androgen receptors (ARs) in their livers. Activated ERs were required for the relatively later initiation of hepatocyte proliferation in females. Using RNA-sequencing (RNA-Seq) analysis, we also identified S100A1 as being involved in sex-biased liver regeneration following PH. S100A1 knockdown inhibited Yap1 activity in larvae and delayed their liver regeneration following chemical-induced injury. Moreover, heterozygous KO of S100A1 inhibited Yap1 activity in adult male zebrafish livers and delayed their liver cell proliferation following PH. Together, these findings revealed the crucial roles played by sex hormone receptors, including ARs and ERs, and the S100A1-Yap1 signaling cascade in determining sex disparity in zebrafish liver cell proliferation following PH.

## RESULTS

### Male zebrafish recover liver mass earlier than female zebrafish following PH through compensatory liver regeneration

**Fig. 1.**
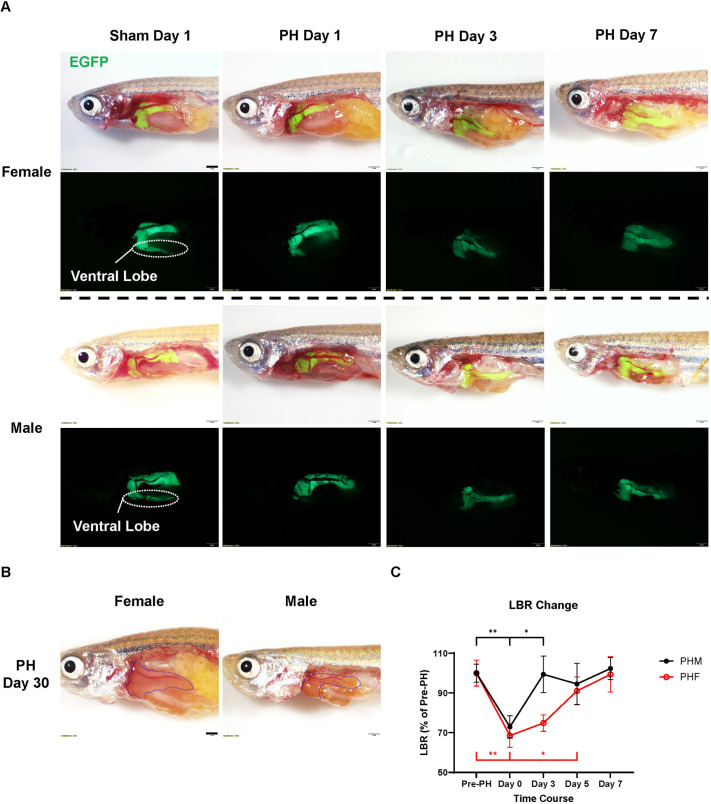
**Compensatory liver regeneration in zebrafish of both sexes following partial hepatectomy (PH).** (A) Representative fluorescence images of EGFP^+^ zebrafish within a week after PH or sham surgery (*n*=3). (B) Representative images of male and female zebrafish 30 days after PH (*n*=3). Yellow dotted lines outline the livers. (C) Changes in the liver-to-body ratio (LBR) of zebrafish of both sexes during PH-induced liver regeneration (*n*=5). Scale bars: 1 mm. *P≤0.05, **P≤0.01 (two-tailed unpaired Student's *t*-test). PHF, female zebrafish with partial hepatectomy; PHM, male zebrafish with partial hepatectomy.

Previously, conflicting results have been reported regarding whether zebrafish can regrow the resected ventral lobe (Dovey et al., 2009; Goessling et al., 2009; Kan et al., 2009; Zhu et al., 2014). Oderberg and Goessling (2021) suggested that only some zebrafish can regrow the resected ventral lobe after 36 days. In the current study, we performed PH on Tg(*fabp10a*:LOXP-EGFP-LOXP-DsRed; TETRE:Cre-ERT2) zebrafish to visualize the shape of the regenerating liver (Li et al., 2019). As shown in Fig. 1A, the resected ventral lobes had no sign of regrowth within 7 days post-PH in both sexes, which is consistent with most previous reports (Kan et al., 2009; Oderberg and Goessling, 2021; Zhu et al., 2014). Later, a long-term experiment was conducted, and we found that neither female nor male zebrafish regrew their resected liver even after 1 month of recovery (Fig. 1B). The regrowth of ventral lobes was not observed in subsequent experiments, suggesting that the zebrafish used in this study underwent compensatory liver regeneration after PH.

The liver-to-body ratio (LBR) is commonly used to indicate changes in the liver mass (Batmunkh et al., 2017; Kan et al., 2009). Based on the LBR of EGFP^+^ zebrafish before and after receiving PH, the recovery of the LBR in zebrafish of both sexes during liver regeneration was measured (Fig. 1C). In male zebrafish, a significant increase in their LBR relative to that on Day 0 was observed on Day 3, which made their LBR comparable with the pre-PH level. By contrast, female zebrafish began their LBR recovery no earlier than 5 days post-PH. By Day 7, both male and female zebrafish achieved full recovery of their LBR. Therefore, male zebrafish recover liver mass earlier than female zebrafish after receiving PH.

### Hepatocyte proliferation and hypertrophy initiate earlier in male zebrafish than in female zebrafish following PH

**Fig. 2.**
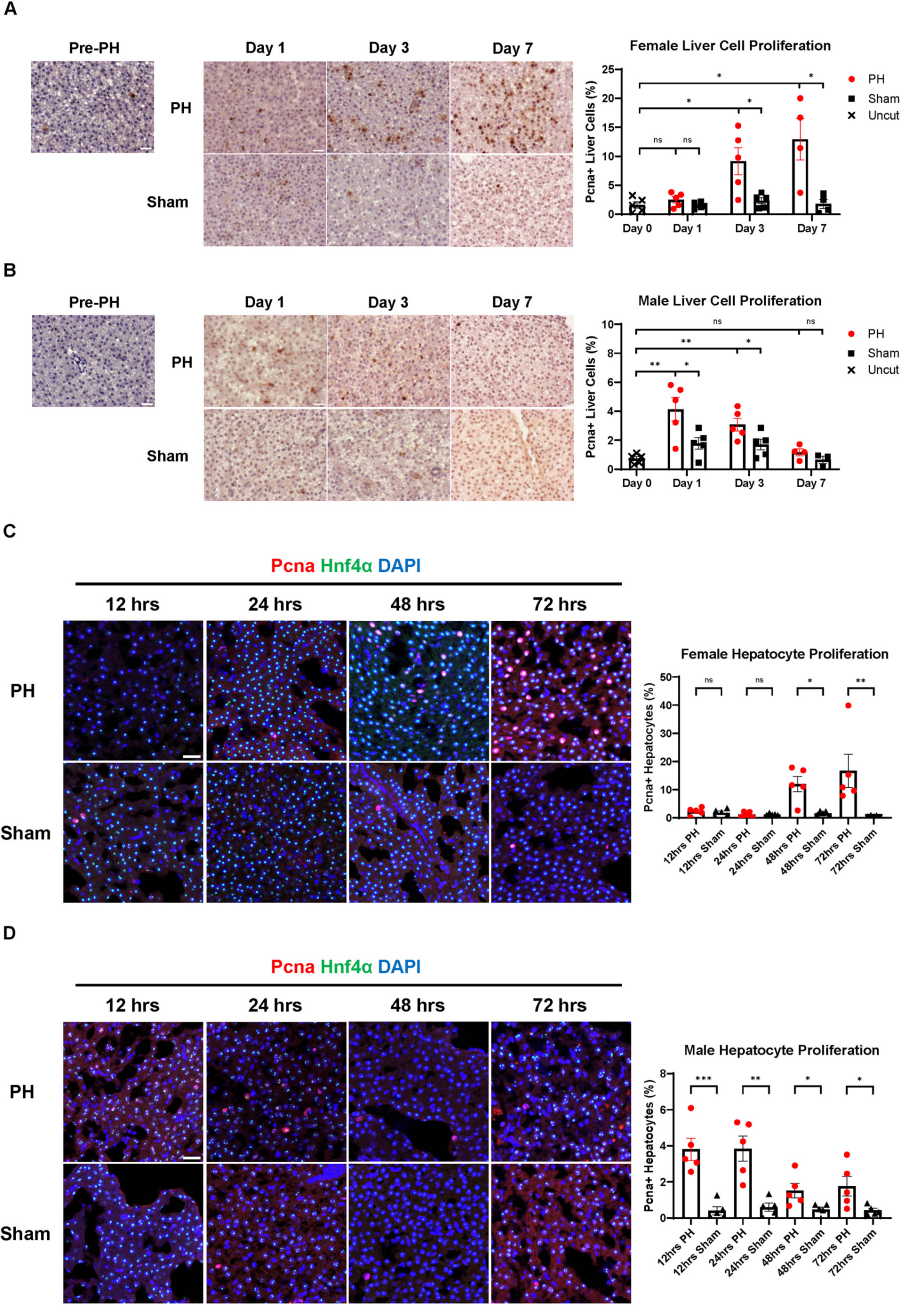
**Sex disparity in liver cell proliferation during PH-induced zebrafish liver regeneration.** (A) Immunohistochemical (IHC) staining of Pcna in female zebrafish livers before and after PH or sham surgery. Quantification of Pcna^+^ proliferating liver cells is shown on the right. Each symbol represents one zebrafish. (B) IHC staining of Pcna in male zebrafish livers before and after PH or sham surgery. Quantification of Pcna^+^ proliferating liver cells is shown on the right. Each symbol represents one zebrafish. **(**C) Immunofluorescence (IF) staining of Pcna and Hnf4α in female zebrafish livers following PH or sham surgery. Quantification of Pcna and Hnf4α double-positive proliferating hepatocytes is shown on the right. Each symbol represents one zebrafish. (D) IF staining of Pcna and Hnf4α in male zebrafish livers following PH or sham surgery. Quantification of Pcna and Hnf4α double-positive proliferating hepatocytes is shown on the right. Each symbol represents one zebrafish. Scale bars: 20 μm. ns, not significant (*P*>0.05); *P≤0.05, **P≤0.01, ***P≤0.001 (two-tailed unpaired Student's *t*-test).

Liver regeneration following PH mainly relies on the proliferation of pre-existing hepatocytes unless the proliferating capability of hepatocytes is inhibited simultaneously (Luther, 2010; Wang et al., 2017). To examine the proliferation level of liver cells in zebrafish of both sexes following PH, we conducted immunohistochemical (IHC) staining of Pcna at 1, 3 and 7 days post-PH (Fig. 2A,B). We found that male zebrafish had significantly increased liver cell proliferation on Day 1 and Day 3, whereas female zebrafish had significantly increased liver cell proliferation on Day 3 and Day 7. Considering the possibility of hepatocyte proliferation initiating before Day 1 and the potential contribution of other cell types towards proliferation, we examined the level of hepatocyte proliferation in wild-type (WT; Singapore indigenous) zebrafish at 12, 24, 48 and 72 h post-PH by co-immunofluorescence (IF) staining of Pcna proliferation marker and Hnf4α hepatocyte marker (Fig. 2C,D). We found that male zebrafish showed significantly increased hepatocyte proliferation as early as 12 h post-PH, whereas female zebrafish did not show an increase until 48 h post-PH. Consistent with the results from IHC and IF imaging, PH induced earlier upregulation of cycle-related genes *ccnd1* and *ccng1* in male livers than in female livers based on real-time quantitative PCR (RT-qPCR) analysis (Fig. S1A,B). In general, the earlier initiation of liver cell proliferation during PH-induced liver regeneration matched the earlier recovery of LBR in male zebrafish.

In addition to hepatocyte proliferation, previous studies have suggested that the enlargement of hepatocytes, termed hepatocyte hypertrophy, contributes to the early recovery of liver mass following liver resection (Miyaoka et al., 2012; Marongiu et al., 2017). To evaluate the sex disparity in liver cell hypertrophy during zebrafish liver regeneration following PH, we performed IF staining of E-cadherin to outline the cell membrane in EGFP^+^ livers, and we measured the area of each hepatocyte based on the staining (Fig. S1C). On the next day of PH, the average size of hepatocytes in males, but not in females, was significantly increased (Fig. S1D). This PH-induced hypertrophy in male zebrafish did not persist but dropped below the baseline 3 days after PH. In female zebrafish, hepatocyte size increased steadily during PH-induced liver regeneration. By Day 7, their livers had significantly larger hepatocytes than those of females before PH. In summary, post-PH, male zebrafish livers showed significant hepatocyte hypertrophy at the early stage of regeneration, which was reversed later, whereas female zebrafish livers showed moderate hepatocyte hypertrophy over the course of regeneration. This earlier hepatocyte hypertrophy in male zebrafish livers could contribute to the earlier recovery of liver mass in male than in female zebrafish following PH.

### Yap1 activity is responsible for the earlier initiation of liver regeneration in male zebrafish than in female zebrafish following PH

**Fig. 3.**
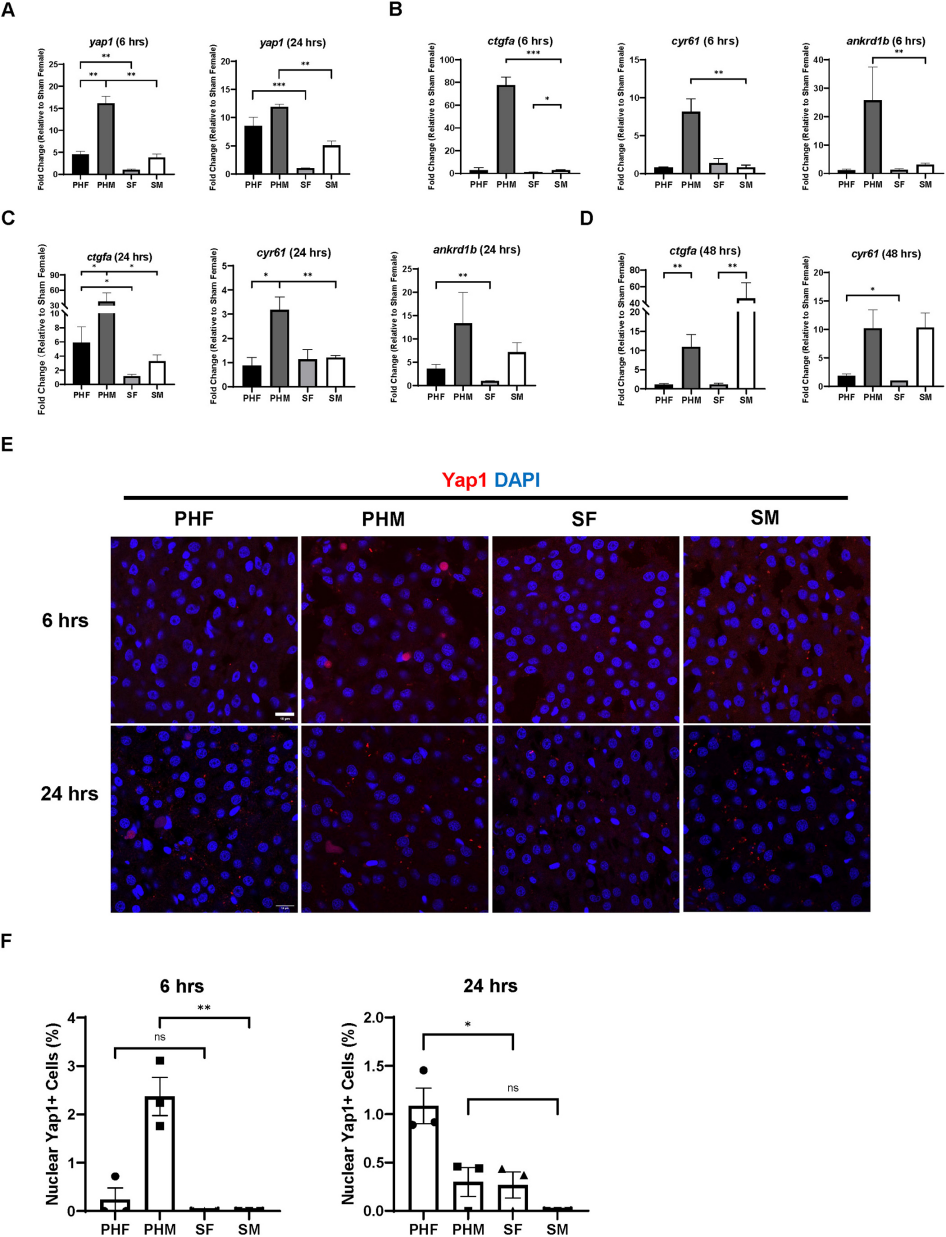
**Earlier initiation of liver regeneration in male zebrafish is associated with earlier Yap1 activation in male livers following PH.** (A) Expression of *yap1* in zebrafish livers of both sexes at 6 h and 24 h post-PH as determined by RT-qPCR (*n*=3). (B,C) Expression of *ctgfa*, *cyr61* and *ankrd1b* in zebrafish livers of both sexes at 6 h (B) and 24 h (C) post-PH as determined by real-time quantitative PCR (RT-qPCR) (*n*=4 for *ankrd1b* at 6 h, *n*=3 for the remaining groups). (D) Expression of *ctgfa and cyr61* in zebrafish livers of both sexes at 48 h post-PH as determined by RT-qPCR (*n*=3). (E) IF staining of Yap1 in the zebrafish liver of both sexes at 6 h and 24 h post-PH. (F) Quantification of Yap1-positive liver cells based on E. Each symbol represents one zebrafish. Scale bars: 10 μm. ns, not significant (*P*>0.05); *P≤0.05, **P≤0.01, ***P≤0.001 (two-tailed unpaired Student's *t*-test).

Because YAP1 activity is known to regulate liver regeneration, we suspected that it might play a role in determining the sex disparity in PH-induced zebrafish liver regeneration. To examine the activity of Yap1 in the zebrafish liver during PH-induced liver regeneration, PH and sham surgery were performed on WT (Tübingen) zebrafish of both sexes. By RT-qPCR analysis, we found that PH induced upregulation of *yap1* in male and female livers within 24 h post-PH (Fig. 3A). Moreover, male zebrafish livers had significantly higher *yap1* expression than female livers at 6 h post-PH. The expression of Yap1 downstream target genes – including *ctgfa*, *cyr61* and *ankrd1b* – was upregulated in male livers at 6 h post-PH (Fig. 3B). Interestingly, at 24 h and 48 h post-PH, these Yap1 downstream target genes started to show significant upregulation in female zebrafish livers, suggesting later activation of Yap1 in females (Fig. 3C,D). Consistent with the results of RT-qPCR, IF staining of Yap1 protein showed that the nuclear Yap1 level in male livers was significantly increased at 6 h post-PH (Fig. 3E,F). By contrast, female zebrafish livers had a significantly increased level of nuclear Yap1 at 24 h post-PH, indicating later activation of Yap1 than in male zebrafish following PH. No increase in nuclear Yap1 was observed in male or female zebrafish livers 72 h after PH (Fig. S1E). Overall, the earlier initiation of liver cell proliferation in male zebrafish following PH was associated with earlier activation of Yap1 in male than in female livers.

**Fig. 4.**
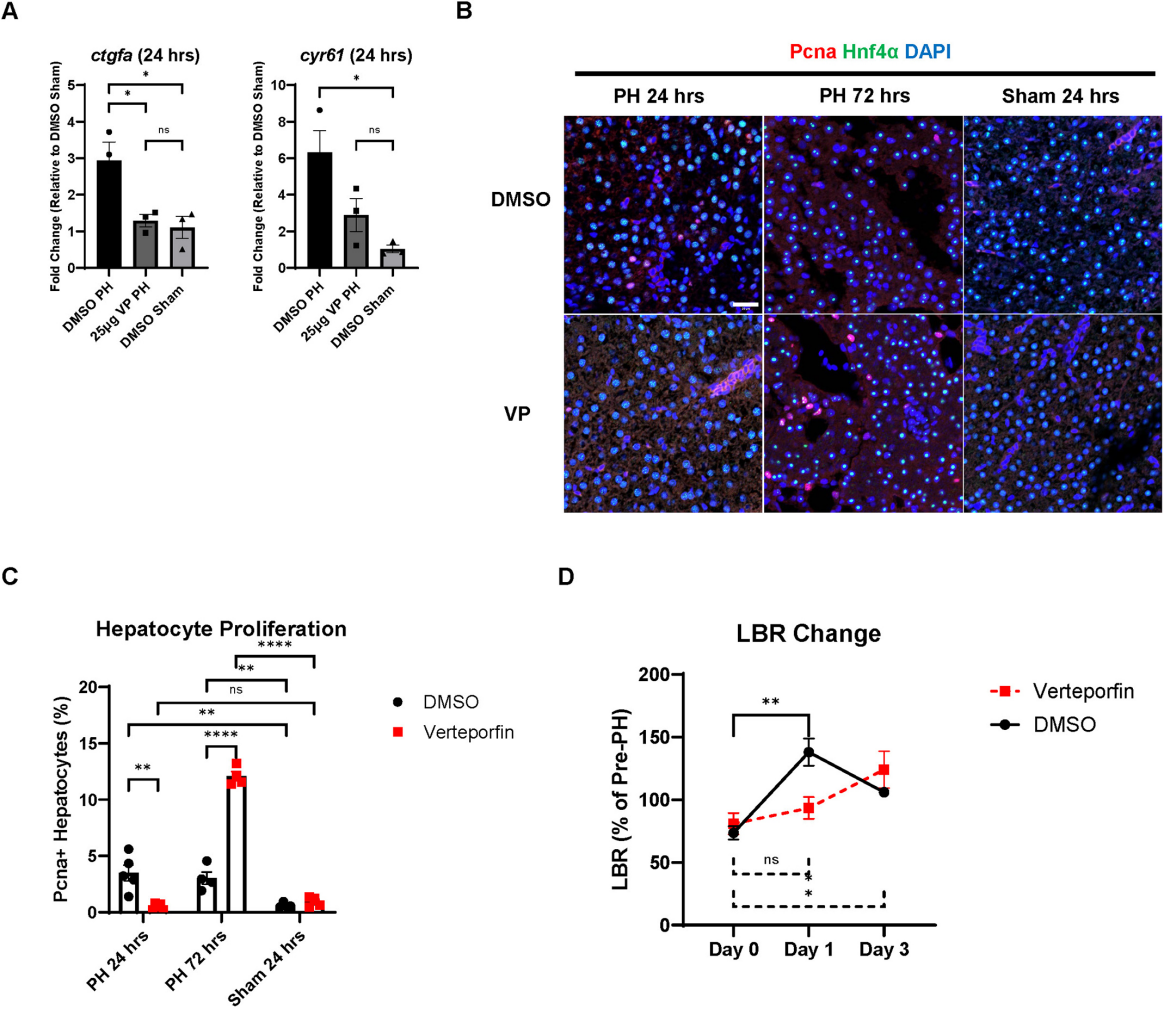
**Effects of Yap1 inhibition on male zebrafish liver regeneration following PH.** (A) Expression of *ctgfa and cyr61* in dimethyl sulfoxide (DMSO)/verteporfin (VP)-pretreated male zebrafish livers at 6 h post-PH/sham surgery as determined by RT-qPCR. Each symbol represents one zebrafish. (B) IF staining of Pcna and Hnf4α in DMSO/VP-pretreated male zebrafish liver following PH or sham surgery. (C) Quantification of Pcna and Hnf4α double-positive proliferating hepatocytes based on B. Each symbol represents one zebrafish. (D) Changes in LBR of DMSO/VP-pretreated male LiPan zebrafish during PH-induced liver regeneration (*n*=3). Scale bar: 20 μm. ns, not significant (*P*>0.05); *P≤0.05, **P≤0.01, ****P≤0.0001 (two-tailed unpaired Student's *t*-test).

To investigate whether the earlier initiation of liver regeneration in male zebrafish requires earlier Yap1 activation, we blocked Yap1 activity in WT (Tübingen) male zebrafish with the Yap1 inhibitor verteporfin (VP) prior to the PH/sham surgeries. Injecting VP into the male zebrafish inhibited the upregulation of Yap1 downstream target genes *ctgfa* (*ccn2a*) and *cyr61* (*ccn1*) in the liver after PH, indicating the successful blockade of Yap1 activity (Fig. 4A). We then checked the levels of hepatocyte proliferation in dimethyl sulfoxide (DMSO)/VP-treated male zebrafish livers during PH-induced liver regeneration based on IF staining of Pcna/Hnf4α. We found that VP treatment inhibited the increase in hepatocyte proliferation at 24 h post-PH while enhancing hepatocyte proliferation at 72 h post-PH, which resulted in a regenerative pattern close to that of female zebrafish (Fig. 4B,C). To examine the effects of Yap1 inhibition on the recovery of LBR during PH-induced liver regeneration, we performed PH on DMSO/VP-treated Tg(*fabp10a*:DsRed; *ela3l*:EGFP) zebrafish (Korzh et al., 2008). Consistent with the inhibited hepatocyte proliferation, the recovery of LBR in VP-treated male LiPan zebrafish after PH was delayed compared with that in the control zebrafish (Fig. 4D). Therefore, Yap1 activation is responsible for the earlier initiation of hepatocyte proliferation in male zebrafish than in female zebrafish following PH.

### ARs regulate the sex disparity in zebrafish liver regeneration following PH by promoting Yap1 activity

**Fig. 5.**
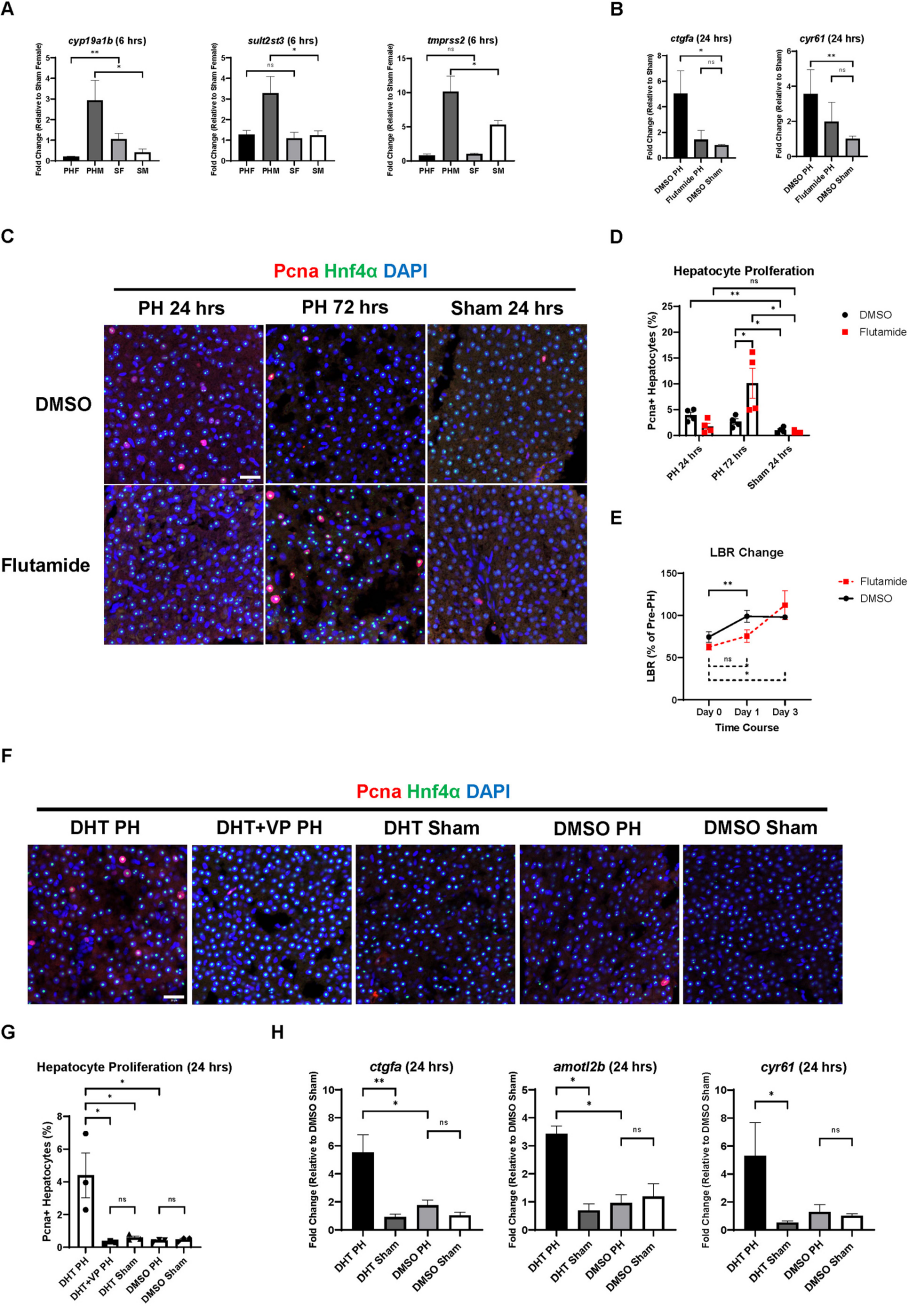
**Androgen receptor (AR) activity regulates male zebrafish liver regeneration following PH in a Yap1-dependent manner.** (A) Expression of *cyp19a1b*, *sult2st3* and *tmprss2* in zebrafish livers of both sexes at 6 h post-PH as determined by RT-qPCR (*n*=3 for *cyp19a1b*, *n*=4 for the remaining groups). (B) Expression of *ctgfa and cyr61* in flutamide/DMSO-treated male zebrafish livers at 24 h post-PH/sham surgery as determined by RT-qPCR (*n*=3). (C) IF staining of Pcna and Hnf4α in DMSO/flutamide-treated male zebrafish livers following PH or sham surgery. (D) Quantification of Pcna and Hnf4α double-positive proliferating hepatocytes based on C. Each symbol represents one zebrafish. (E) Changes in LBR of DMSO/flutamide-pretreated LiPan male zebrafish during PH-induced liver regeneration (*n*=4). (F) IF staining of Pcna and Hnf4α in dihydrotestosterone (DHT)/DHT+VP/DMSO-pretreated zebrafish livers of both sexes at 24 h post-PH. (G) Quantification of Pcna and Hnf4α double-positive proliferating hepatocytes based on F. Each symbol represents one zebrafish. (H) Expression of *ctgfa*, *amotl2b* and *cyr61* in DHT/DMSO-pretreated male zebrafish livers at 24 h post-PH as determined by RT-qPCR (*n*=3). Scale bars: 20 μm. ns, not significant (*P*>0.05); *P≤0.05, **P≤0.01 (two-tailed unpaired Student's *t*-test).

ARs are major sex hormone receptors that regulate the development and maintenance of male-associated phenotypes. In the rat PH model, inhibiting AR activity delayed liver regeneration (Tsukamoto and Kojo, 1990). Based on the results from RT-qPCR, the expression of AR-responsive genes – including *cyp19a1b*, *sult2st3* and *tmprss2* – increased significantly in male zebrafish livers, but not in female livers, at 6 h post-PH (Fig. 5A). To investigate the function of ARs in the sex disparity in zebrafish liver regeneration, we exposed WT (Tübingen) male zebrafish to flutamide, an AR antagonist, before and after the PH/sham surgeries. As shown in Fig. 5B, blocking AR activity with flutamide inhibited the upregulation of the Yap1 downstream targets *ctgfa* and *cyr61* in male zebrafish livers at 24 h post-PH. The initiation of hepatocyte proliferation in the flutamide-treated male zebrafish was also delayed from 24 h post-PH to 72 h post-PH (Fig. 5C,D). In addition, blocking AR activity with flutamide delayed the recovery of LBR following PH in LiPan male zebrafish (Fig. 5E). Therefore, AR activation is required for the earlier initiation of hepatocyte proliferation in male zebrafish than in female zebrafish following PH.

To further examine how ARs regulate the sex disparity in zebrafish liver regeneration, we used an AR agonist, dihydrotestosterone (DHT), to treat female WT (Tübingen) zebrafish prior to PH. In contrast to the effect of flutamide, DHT treatment facilitated the initiation of hepatocyte proliferation in female zebrafish liver regeneration, with a significant increase in hepatocyte proliferation at 24 h post-PH (Fig. 5F,G). Intriguingly, this facilitating effect of DHT on female hepatocyte proliferation following PH could be abolished by blocking Yap1 activity with VP. Previous studies have reported that ARs can bind with YAP1 and promote its nuclear translocation in prostate cancer cells to enhance cell survival (Cinar et al., 2020; Kuser-Abali et al., 2015). In our experiments, DHT treatment induced upregulation of the Yap1 downstream targets *ctgfa*, *amotl2b* and *cyr61* in female zebrafish livers at 24 h post-PH (Fig. 5H). This was consistent with the earlier observed inhibition of expression of Yap1 downstream targets following flutamide treatment. Together, these results suggest that AR activity can regulate the sex disparity in PH-induced zebrafish liver regeneration through promoting Yap1 activity.

### ER activity regulates the pattern of female zebrafish liver regeneration following PH

Some studies have attributed the sex disparity in liver regeneration following PH to high ER activity in females (Batmunkh et al., 2017; Kao et al., 2017; Uebi et al., 2015). To confirm whether ERs are required for the relatively late initiation of female liver regeneration following PH, female WT (Tübingen) zebrafish were exposed to the ER antagonist ICI 182, 780 (ICI) from 48 h prior to the PH/sham surgeries. IF staining of Pcna proliferation marker and Bhmt hepatocyte marker was conducted to measure the level of hepatocyte proliferation (Fig. S2A). We found that inhibiting ERs with ICI advanced the initiation of hepatocyte proliferation in female zebrafish from 72 h post-PH to 24 h post-PH, which resulted in a pattern similar to that of liver regeneration in male zebrafish (Fig. S2B). In addition, the recovery of LBR in female LiPan zebrafish at 24 h post-PH was enhanced by ICI treatment (Fig. S2C). Notably, ICI treatment did not alter the expression of Yap1 downstream targets *ctgfa* and *cyr61* in female livers at 24 h post-PH (Fig. S2D). This suggests that, unlike AR regulation of liver regeneration, ERs do not regulate liver regeneration by affecting Yap1 activity. To confirm whether the effect of ICI treatment relies on blocking the interaction between ER and estrogen, a nonsteroidal aromatase inhibitor, fadrozole, was used to block the production of estrogen in WT (Tübingen) female zebrafish during PH-induced liver regeneration. Fadrozole treatment facilitated the initiation of hepatocyte proliferation in female zebrafish following PH, which was consistent with the results from the ER blockade experiments (Fig. S2E,F). Therefore, the activity of ERs is responsible for the later initiation of hepatocyte proliferation following PH in female zebrafish than in male zebrafish.

### PH induces transcriptomic changes differently between male livers and female livers

**Fig. 6.**
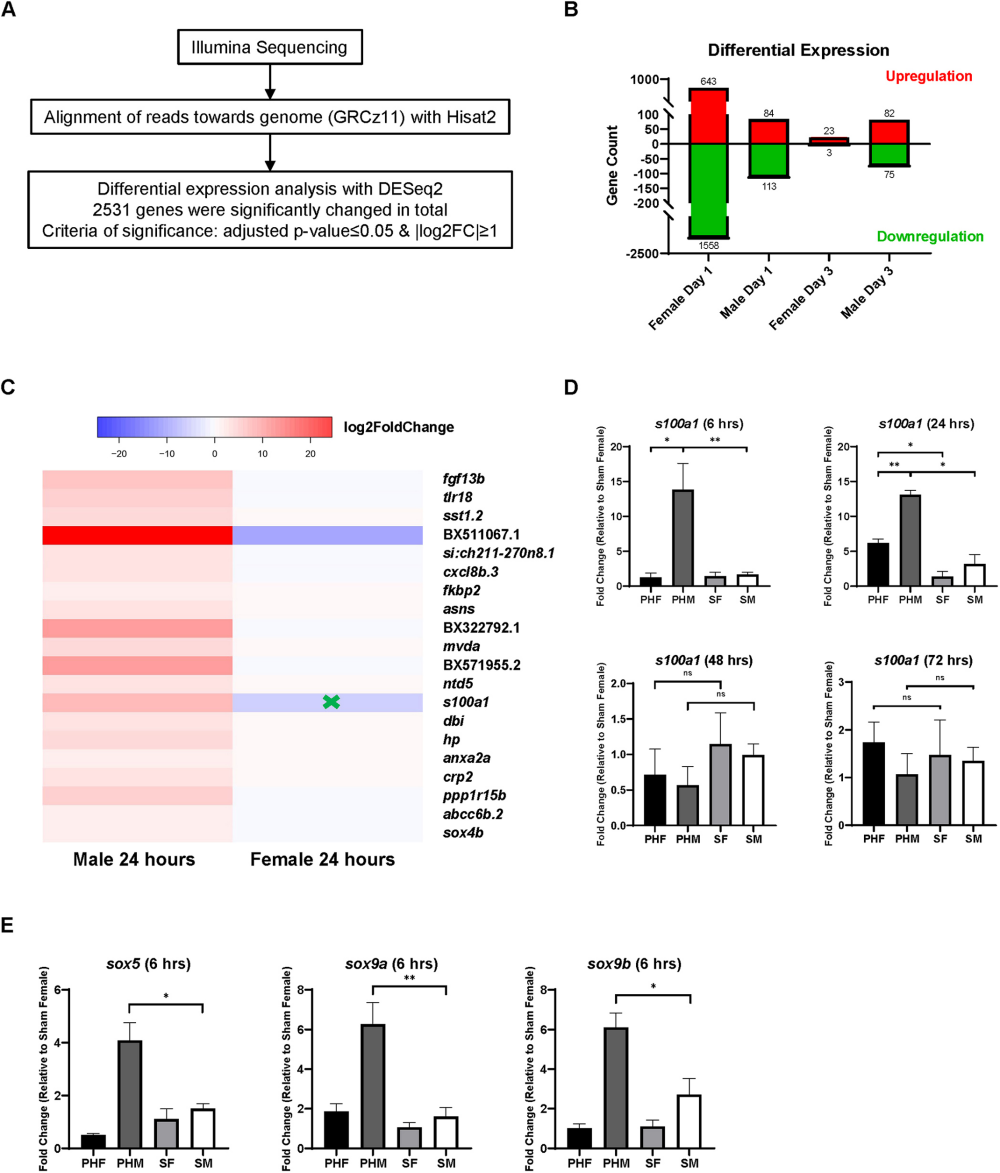
**Sex disparity in transcriptomic regulation during PH-induced zebrafish liver regeneration.** (A) Workflow of differentially expressed gene analysis based on the RNA-sequencing results of PH-treated zebrafish livers of both sexes versus the corresponding sham-treated livers on Day 1 and Day 3. log2FC, log2 fold change. (B) Quantification of the significantly upregulated/downregulated genes identified in A. (C) The top 20 genes that were significantly upregulated only in male livers at 24 h post-PH. The green cross indicates significant downregulation. (D) Expression of *s100a1* in zebrafish liver of both sexes following PH/sham surgeries as determined by RT-qPCR (*n*=3). (E) Expression of *sox5*, *sox9a* and *sox9b* in zebrafish liver of both sexes at 6 h post-PH as determined by RT-qPCR (*n*=3 for *sox5*, *n*=4 for the remaining groups). ns, not significant (*P*>0.05); *P≤0.05, **P≤0.01 (two-tailed unpaired Student's *t*-test).

To identify potential regulators of sex disparity in PH-induced liver regeneration, we conducted RNA-Seq with the WT (Singapore indigenous) zebrafish liver tissues collected on Day 1 and Day 3 following PH. By comparing the transcriptome in the PH livers and the sham surgery-treated livers, 2531 differentially expressed genes (DEGs) during PH-induced liver regeneration were identified (Fig. 6A). As shown in Fig. 6B, the numbers of genes that were significantly deregulated by PH were markedly different between male and female liver. Female zebrafish had 2181 DEGs in the liver after PH on Day 1, whereas male zebrafish had only 197 DEGs. On Day 3, 157 DEGs were found in the male livers, and only 26 DEGS were found in the female livers. Moreover, DEGs in different sex groups at different time points during the PH-induced liver regeneration showed very limited overlap among each other (Fig. S3A,B).

Considering the earlier initiation of liver regeneration in male than in female zebrafish following PH, we focused on genes that were significantly upregulated in the liver of one sex, but not upregulated [log2 fold change (FC)<1] in the liver of the other sex, on Day 1. Table S1 lists the top 20 genes that were most significantly upregulated in the male and female liver on Day 1. Of these genes, *BX511067.1* had the strongest upregulation in the PH male livers. However, *BX511067.1* is a gene identified recently, and its function is completely unknown. This made it difficult to predict its potential role in liver regeneration. Another gene of interest was *s100a1*, which was the only gene that was significantly upregulated in the male zebrafish liver while being downregulated in the female zebrafish liver on Day 1 (Fig. 6C). To verify the male-predominant upregulation of *s100a1* in the zebrafish liver at the early stage of liver regeneration, we performed RT-qPCR analysis with WT (Tübingen) zebrafish liver samples collected at 6 h, 24 h and 48 h post-PH. We found that male zebrafish livers showed *s100a1* upregulation since 6 h post-PH, whereas female zebrafish livers showed this upregulation only after 24 h (Fig. 6D). In addition, the level of *s100a1* expression in the PH male zebrafish livers was significantly higher than that in the PH female livers. The upregulation of *s100a1* in female and male livers disappeared by 48 h post-PH. The expression of S100A1 can be induced by SOX9, which is related to sex determination and reversal, and another SOX family member, SOX5, can further enhance the SOX9-induced upregulation of S100A1 (Saito et al., 2007). In our experiments, both *sox5* and *sox9* were found to be upregulated only in male livers at 6 h post-PH (Fig. 6E). Because the sex disparity in the upregulation of *s100a1*, *sox5* and *sox9* matched that of liver regeneration, we hypothesized that S100A1 plays a role in the sex disparity in PH-induced zebrafish liver regeneration.

To investigate the sex disparity in the regulation of signaling pathways and other biological functions during PH-induced zebrafish liver regeneration, we performed gene set enrichment analysis (GSEA) of hallmark gene sets with the RNA-Seq results of the regenerating livers of female and male zebrafish. The results were categorized based on the previous study by Liberzon et al. (2015). We found that a major proportion of hallmark pathways were affected differentially by PH between males and females (Fig. S3C,D). For example, the pathways of unfolded protein response and mTORC1 signaling were upregulated in male zebrafish livers on Day 1 but were not upregulated in female livers until Day 3, which matched the sex disparity in liver regeneration after PH. The activation of the unfolded protein response might be caused by increased protein production associated with cell proliferation, while mTORC1 signaling has been reported to regulate hepatocyte hypertrophy during PH-induced liver regeneration (Fouraschen et al., 2013; Haga et al., 2005). Many pathways, such as those in the immune, metabolic and proliferation categories, were regulated differently by PH on Day 3 relative to Day 1 in zebrafish livers. This suggests that activation of those pathways could play a temporal role in liver regeneration and that their regulation could represent regeneration progresses. Most pathways under the immune category and proliferation category were significantly upregulated in the female zebrafish liver on Day 1 and Day 3, respectively. The upregulation of proliferation-related pathways in the female zebrafish liver on Day 3 matched the significantly increased hepatocyte proliferation in females at the same time point following PH. By contrast, we did not find an overall activation of proliferation-related pathways in the male zebrafish liver on Day 1, which was inconsistent with the observed earlier hepatocyte proliferation in males than in females during PH-induced liver regeneration. Considering that the initiation of male hepatocyte proliferation can be traced as early as 12 h post-PH, these pathways could possibly be activated in the male liver before the examined time points. To support that, early E2 response was upregulated in the female livers before Day 1, whereas male livers had upregulation of the late E2 response at that time point, implying that activation of the early E2 response in male livers could have already finished by Day 1. In addition to the sex disparity, PH-induced deregulation of hallmark pathways in zebrafish livers changed over time. Many pathways, such as the pathways under the categories of immune, metabolic and proliferation, were regulated reversely by PH on Day 3 compared with their regulation on Day 1 in male and female zebrafish. This phenomenon suggested that those pathways could play a temporal role in liver regeneration and that their activities might represent the progress of regeneration (e.g. initiation or termination). Overall, the differential regulation of genes and pathways between male livers and female livers following PH provided us with candidates potentially responsible for the observed sex disparity in PH-induced zebrafish liver regeneration.

### *s100a1* knockdown delays liver expansion during zebrafish larvae development and liver regeneration via inhibiting Yap1 activity

Because the function of S100A1 in the normal liver has not been studied before, we used the morpholino (MO) targeting the start codon of *s100a1* (MO-*s100a1*-ATG) to investigate whether *s100a1* knockdown affects liver development and regeneration in Tg(*fabp10a*:CFP-NTR) zebrafish larvae (Choi et al., 2014). As shown in Fig. S4A, knockdown of *s100a1* significantly reduced the size of the liver in nitroreductase (NTR)^+^ larvae at 5 days post fertilization (dpf). By 6 dpf, the size of the livers in the *s100a1*-knockdown larvae became comparable with that of the control larvae probably due to the loss of MO activity (Fig. S4B). Consistent with the impaired liver expansion, reduced expression of cell cycle-related genes *ccnd1* and *cdk1* was found in the *s100a1*-knockdown group at 4.5 dpf (Fig. S4C).

**Fig. 7.**
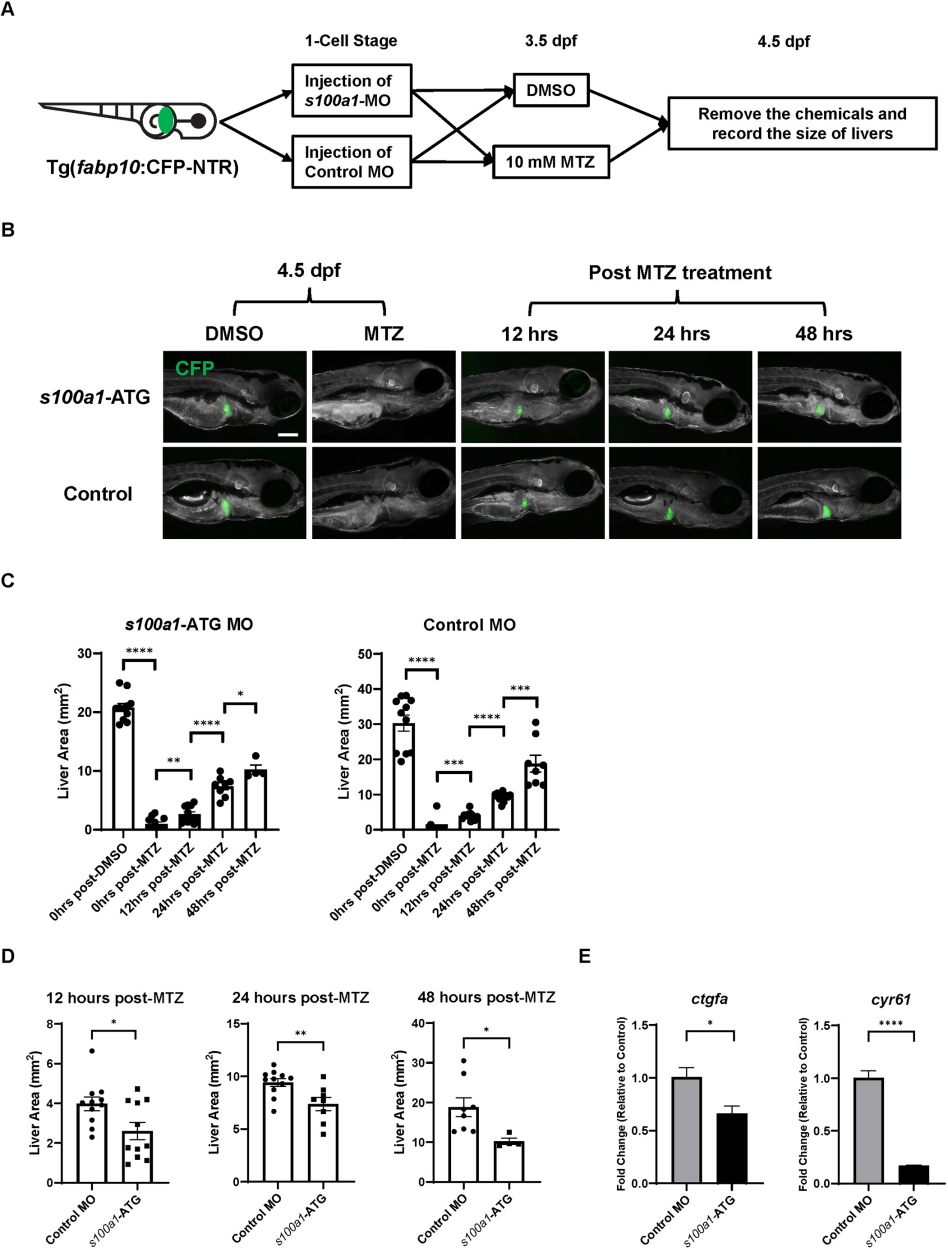
**Effects of *s100a1* knockdown on liver expansion in zebrafish larvae during chemical injury-induced liver regeneration.** (A) Experimental design for investigating the effects of *s100a1* knockdown on liver regeneration of NTR^+^ larvae following metronidazole (MTZ)-induced hepatocyte ablation. NTR^+^ larvae injected with morpholino (MO) were exposed to 10 mM MTZ or DMSO for 24 h from 3.5 days post fertilization (dpf). (B) Fluorescence images of MO-injected NTR^+^ larvae following 24 h of DMSO or MTZ treatment. (C,D) Measurement and comparison of the area of CFP^+^ livers in MO-injected NTR^+^ larvae based on B. Each symbol represents one larva. (E) Expression of *ctgfa and cyr61* in MO-injected NTR^+^ larvae 24 h after MTZ treatment as determined by RT-qPCR (*n*=3). Each sample used for quantitative PCR contained cDNA from 15 larvae. Scale bar: 200 μm. *P≤0.05, **P≤0.01, ***P≤0.001, ****P≤0.0001 (two-tailed unpaired Student's *t*-test).

With NTR^+^ zebrafish, we were able to induce hepatocyte ablation with metronidazole (MTZ) for studying the effect of *s100a1*-knockdown on chemical-induced liver regeneration (Fig. 7A). Twenty-four hours of MTZ treatment significantly reduced the liver size of NTR^+^ larvae, and then the liver sizes in both the *s100a1*-knockdown larvae and control larvae gradually recovered over time (Fig. 7B,C). The *s100a1*-knockdown larvae regenerated significantly smaller livers than did the control larvae within 48 h after MTZ-induced injury (Fig. 7D). Therefore, the expansion of the liver during embryonic development and liver regeneration in zebrafish larvae required the participation of S100A1. To examine whether *s100a1* knockdown affects Yap1 activity, we performed RT-qPCR analysis of the expression of Yap1 downstream targets in NTR^+^ larvae after 24 h of MTZ treatment. We found that knockdown of *s100a1* significantly reduced the expression of Yap1 downstream targets, including *ctgfa* and *cyr61*, in NTR^+^ larvae (Fig. 7E). This result suggests that S100A1 can regulate liver expansion in zebrafish larvae by promoting Yap1 activity.

### Reduced *s100a1* expression delays PH-induced liver regeneration in male zebrafish by inhibiting Yap1 activity

To confirm whether the sex-biased liver regeneration in zebrafish following PH requires the expression of *s100a1*, we generated an *s100a1*-KO zebrafish strain with CRISPR/Cas9-mediated large-fragment deletion. Two guide RNAs (gRNAs) were designed to target the 5′ and 3′ terminals of the *s100a1* coding region and microinjected into WT (AB) zebrafish embryos together with the Cas9 protein (Fig. S5A). This resulted in the deletion of the whole translated region of *s100a1*. By PCR and agarose gel electrophoresis, we confirmed that heterozygous and homozygous *s100a1*-KO zebrafish have the short allele resulting from the deletion in their liver genomes (Fig. S5B). In addition, full-length *s100a1* was not present in the liver genome of homozygous KO fish, indicating complete loss of the full-length allele. After sequencing the short deletion allele, we confirmed that the targeted region was deleted completely in the genome of *s100a1*-KO zebrafish (Fig. S5C). In addition, the expression level of *s100a1* in the *s100a1*^+/−^ male livers was significantly lower than that in the livers of their WT siblings, suggesting that the heterozygous KO induced haploinsufficiency of *s100a1* (Fig. S5D). However, it seems that liver growth in adult zebrafish was not affected by *s100a1* haploinsufficiency as the LBR in *s100a1*^+/−^ males was comparable with that in WT males (Fig. S5E).

**Fig. 8.**
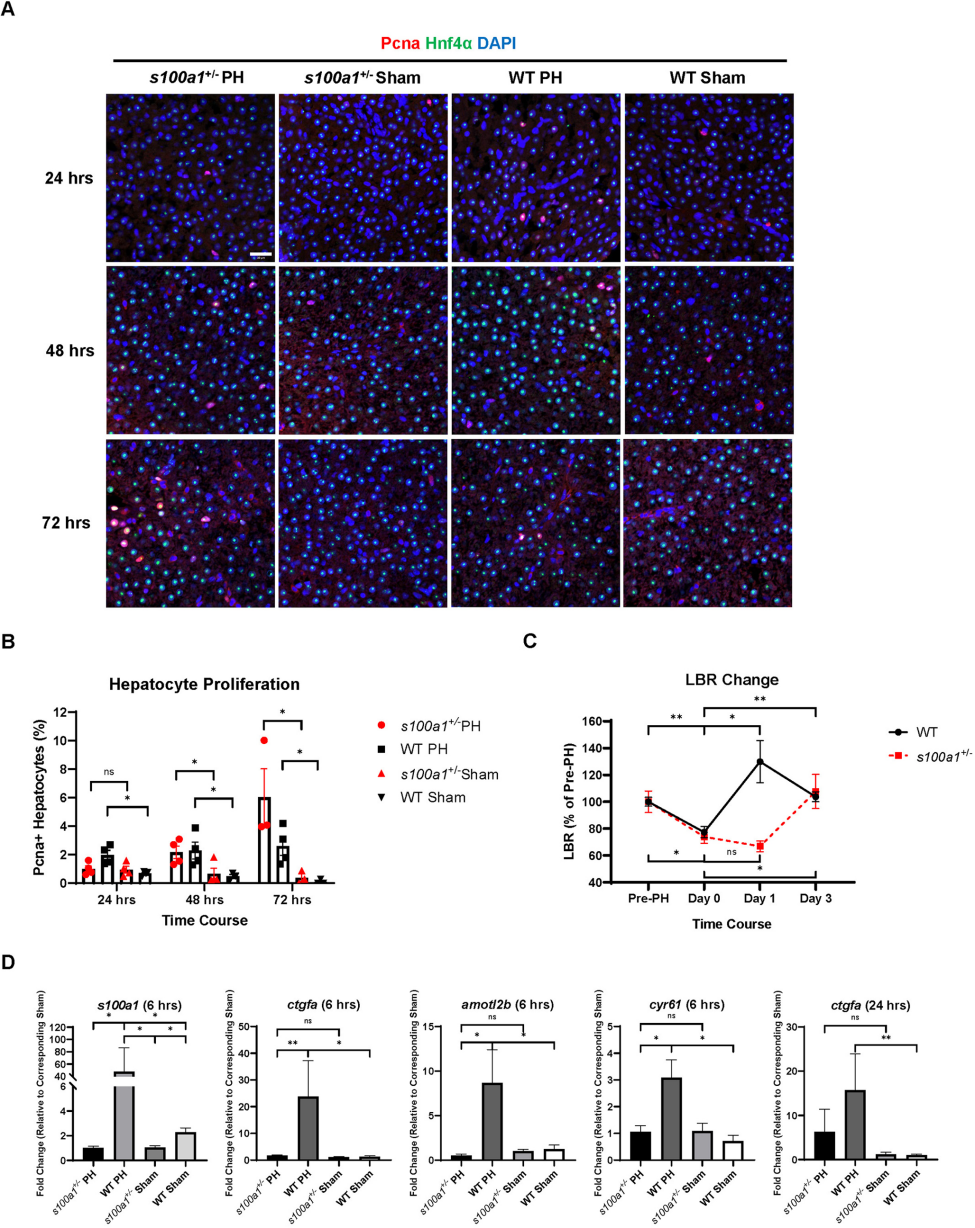
**Effects of *s100a1* haploinsufficiency on male zebrafish liver regeneration following PH.** (A) IF staining of Pcna and Hnf4α in *s100a1*^+/−^ and wild-type (WT) male zebrafish livers following PH or sham surgery. (B) Quantification of Pcna and Hnf4α double-positive proliferating hepatocytes based on A. Each symbol represents one zebrafish. (C) LBR changes in *s100a1*^+/−^ males compared with WT males during PH-induced liver regeneration (*n*=4). (D) Expression of *s100a1*, *ctgfa*, *amotl2b* and *cyr61* in *s100a1*^+/−^ and WT livers at 6 h or 24 h post-PH as determined by RT-qPCR (*n*=3). Scale bar: 20 μm. ns, not significant (*P*>0.05); *P≤0.05, **P≤0.01 (two-tailed unpaired Student's *t*-test).

With this newly generated zebrafish mutant strain, we compared PH-induced liver regeneration between *s100a1*^+/−^ male zebrafish and WT (AB) male zebrafish. Based on the results from IF imaging, *s100a1*^+/−^ male zebrafish showed inhibited hepatocyte proliferation compared with their WT siblings at 24 h post-PH, which indicates delayed initiation of liver regeneration (Fig. 8A,B). Furthermore, the recovery of LBR was delayed in *s100a1*^+/−^ males compared with WT males following PH (Fig. 8C). Because we proposed that S100A1 regulates zebrafish liver regeneration by regulating Yap1 activity, we examined the expression level of *s100a1* and Yap1 downstream target genes in the *s100a1*^+/−^ male livers and WT male livers after PH. As expected, the upregulation of *s100a1* at the early phase of PH-induced liver regeneration was inhibited in the *s100a1*^+/−^ male livers (Fig. 8D). The expression levels of Yap1 downstream targets *ctgfa*, *amotl2b* and *cyr61* were upregulated in the WT livers but not in the *s100a1*^+/−^ livers at 6 h post-PH. The inhibition of the upregulation of *ctgfa* in the *s100a1*^+/−^ livers continued until 24 h post-PH. In addition, *s100a1*^+/−^ male zebrafish showed no hepatocyte hypertrophy within 72 h following PH, indicating that hepatocyte hypertrophy at the early stage of regeneration requires S100A1 (Fig. S6A,B). Therefore, S100A1 drives the earlier initiation of liver regeneration in male zebrafish following PH via regulating hepatocyte hypertrophy and promoting Yap1-mediated hepatocyte proliferation.

## DISCUSSION

Zebrafish have been widely used in modeling human diseases including tissue regeneration owing to their physiological and molecular similarities with humans, high regenerative capability and amenability for *in vivo* imaging. The existence of sex disparity in liver regeneration following PH has been well demonstrated in the clinic and in animal models (Batmunkh et al., 2017; Birrer et al., 2022; Wang et al., 2013). In the current study, we found that male zebrafish began liver regeneration earlier than female zebrafish following PH in terms of liver cell proliferation and LBR recovery. The earlier liver cell proliferation observed in male zebrafish is consistent with previous reports of male zebrafish recovering LBR earlier than female zebrafish following PH (Kan et al., 2009). Compared with the results from previous studies using rodent PH models, the male-biased liver regeneration observed in zebrafish is consistent with that in mice (Wang et al., 2013; Zhou et al., 2016) but contradictory to that in rats (Batmunkh et al., 2017). Currently, there is an absence of clinical data on the sex disparity in liver regeneration following PH. A recent study showed that female patients have faster liver regeneration than male patients, but the data were obtained from patients who received liver partition and portal vein ligation rather than liver resection (Birrer et al., 2022). Therefore, data from the current study conducted in zebrafish, together with the earlier works conducted in rodents, may provide valuable references for improving the clinical application of PH-based therapies.

After liver resection, mammal livers enlarged the remnant to compensate the lost liver mass rather than regenerating the resected liver lobes. Nevertheless, the terms ‘liver regeneration’ and ‘compensatory liver regeneration’ have traditionally been used to describe this process in the literature (Abu Rmilah et al., 2019; Michalopoulos, 2007). In the current study, we found that both male and female zebrafish recovered their LBR by 7 days after PH without regrowing their resected ventral lobes. This compensatory regeneration in the short term is consistent with mammalian liver regeneration and many previous reports on zebrafish liver regeneration (Kan et al., 2009; Sadler et al., 2007; Zhu et al., 2014). For long-term recovery, Kan et al. (2009) suggested no regrowth of ventral lobes in zebrafish up to 6 months post-PH, while Zhu et al. (2014) observed regrowth of ventral lobes within 60 days. The most recent study by Oderberg and Goessling (2021) showed that zebrafish livers have the capability for both epimorphic and compensatory regeneration. In their study, only one transgenic zebrafish line was used, but only some of the fish could regrow their resected liver lobes within 36 days post-PH. It is unclear whether this variation in the capability of regeneration is due to subtle differences in the genetics. In the current study, we did not observe any sign of regrowth even after 30 days.

Although male zebrafish showed a significant increase in hepatocyte proliferation as early as 12 h post-PH, the absolute level of proliferation was only ∼5%. In the current study, the LBR of male zebrafish showed full recovery as early as 24 h after 30% of PH. Unless the speed of liver cell mitosis in adult zebrafish is comparable with that in embryonic development, it would be difficult for male zebrafish to recover their LBR in such a short period with only a low level of hepatocyte proliferation. One explanation could be that male zebrafish compensate for the lost liver mass rapidly not only by hepatocyte proliferation but also by hepatocyte hypertrophy at the early stage of liver regeneration. Hepatocyte hypertrophy has been suggested to contribute to liver regeneration together with hepatocyte proliferation in rodent PH models (Miyaoka et al., 2012; Marongiu et al., 2017). In our experiments, significant hepatocyte hypertrophy occurred in the male zebrafish liver at 1 day post-PH but regressed at the later time points. The hepatocyte hypertrophy during PH-induced liver regeneration is mediated by mTORC1 signaling activation (Fouraschen et al., 2013; Haga et al., 2005). Consistently, our GSEA results also showed that the mTORC1 pathway was significantly upregulated in the male zebrafish liver at Day 1 post-PH. Whether hepatocyte hypertrophy is required for the earlier LBR recovery of male livers could be a focus for future studies.

The Hippo-YAP1 signaling pathway controls organ regeneration and tumorigenesis by regulating cell differentiation and proliferation (Hansen et al., 2015; Kowalczyk et al., 2022). Blocking YAP1 activity impaired hepatocyte proliferation and liver mass recovery in mice following PH, demonstrating that YAP1 activity is important to PH-induced liver regeneration (Liu et al., 2019; Lu et al., 2018). In the current study, we have found that the earlier hepatocyte proliferation and LBR recovery in male zebrafish following PH required earlier activation of Yap1 in male livers. This result suggests, for the first time, the role played by Yap1 activity in regulating the sex disparity in liver regeneration. The uptake of testosterone in the rat liver after PH suggests that ARs participate in regulating liver regeneration (Yamaguchi et al., 2001). However, the specific roles played by ARs in liver regeneration are poorly understood. In the current study, we found that ARs regulated the earlier initiation of hepatocyte proliferation and LBR recovery in male than in female zebrafish after PH. ARs have been found to bind with YAP1 in prostate cancer cells and enhance its activity by facilitating its nuclear translocation (Cinar et al., 2020; Kuser-Abali et al., 2015). In our experiments, AR activity regulated the initiation of hepatocyte proliferation following PH in a Yap1-dependent manner. Thus, ARs regulate the sex disparity in zebrafish liver regeneration, at least partially, through interacting with Hippo-Yap1 signaling.

In zebrafish, estrogen promotes hepatocyte proliferation and regulates the sex disparity in adult liver size during development (Chaturantabut et al., 2019). Notably, the promoting effect of estrogen on zebrafish liver growth is mediated by G protein-coupled estrogen receptors (GPERs) rather than ERs. In a previous study, inhibiting GPER activity, but not ER activity, during estrogen treatment abolished the estrogen-induced liver growth in zebrafish, whereas activating GPER alone induced a comparable level of hepatocyte proliferation as estrogen treatment (Chaturantabut et al., 2019). In the current study, inhibiting ER activity in female zebrafish livers with ICI promoted their hepatocyte proliferation and LBR recovery at the early stage following PH. As ICI is an inhibitor targeting ERs, this result does not necessarily conflict with the promoting effect of estrogen on hepatocyte proliferation mediated by GPER. However, ICI treatment has been reported to attenuate estrogen-induced liver cell proliferation in rats, which conflicts with the idea that estrogen promotes hepatocyte proliferation not through activating ERs (Batmunkh et al., 2017). Future studies may need to confirm the specificity of ICI on ERs and the effect of ER activation alone on liver regeneration without potential interference from GPER activity.

According to our RNA-Seq analysis of the regenerating zebrafish livers, the regulation of genes during PH-induced liver regeneration was very different between male zebrafish and female zebrafish. We found a relatively small number of significantly deregulated genes in male livers compared with female livers on Day 1 after PH. This result seems inconsistent with the earlier responses of male zebrafish than female zebrafish towards PH. It is likely that the time point we chose to collect samples was not early enough to catch rapid changes in RNA expression. We identified S100A1 as a potential regulator responsible for sex disparity in zebrafish liver regeneration after PH because it shows a male-biased expression significantly at the early stage of regeneration. As a calcium-binding protein, the main function of S100A1 is regulating the release and uptake of calcium in the smooth reticulum in a calcium level-dependent manner (Scott and Kekenes-Huskey, 2016). Recently, Wang et al. (2021) have demonstrated that S100A1 can regulate the Hippo-YAP1 signaling pathway in papillary thyroid carcinoma cells to promote cell proliferation. According to their study, knockdown of S100A1 can promote the phosphorylation of YAP1, which inactivates YAP1 and results in reduced nuclear YAP1 level and inhibited cell growth. In the current study, deficiency in S100A1 reduced Yap1 activity and impaired liver regeneration in both larval and adult zebrafish. Furthermore, the direct transcription regulators of S100A1, including Sox5 and Sox9, showed male-biased upregulation in zebrafish livers at the early stage of PH-induced regeneration. Based on these results, we propose a signaling cascade regulating the sex disparity of zebrafish liver regeneration following PH: PH induces expression of Sox5 and Sox9 in male zebrafish livers; the increased level of Sox5 and Sox9 further induce the transcription of S100A1, resulting in earlier upregulation of S100A1 in male livers than females; subsequently, S100A1 promotes the activation of Yap1 through inhibiting its phosphorylation; and the activated Yap1 then induces the expression of downstream genes and initiates liver cell proliferation. Exactly how S100A1 regulates Yap1 phosphorylation requires further investigation.

Previous studies have demonstrated the function of S100A1 and ARs in regulating YAP1 activity (Cinar et al., 2020; Wang et al., 2021). In the current study, we confirmed that both ARs and S100A1 regulate sex disparity in liver regeneration by regulating Yap1 activity. These results seem to suggest a potential interaction between sex hormone receptors and S100A1. However, there are not yet any studies on this topic. In addition, *s100a1* has not been identified as an estrogen-responsive gene or an androgen-responsive gene (Jin et al., 2013; Nishi et al., 2022). Therefore, no direct links can be established between sex hormone receptors and S100A1 except that both regulate Yap1 signaling activity.

In addition to *s100a1*, some other genes that can promote cell proliferation – such as *anxa2a*, *fgf13b* and *postnb* – were also upregulated only in the male zebrafish livers on Day 1 following PH (Li et al., 2021; Lu et al., 2020; Wu et al., 2018). Of them, *postnb* encodes an isoform of periostin, and periostin has been found to be required for competent liver regeneration after PH in mice (Wu et al., 2018). Knockout of *Postn* resulted in impaired liver regeneration in terms of hepatocyte proliferation, LBR recovery and immune responses following PH in mice. Because periostin was mainly produced by hepatic stellate cells (HSCs) in the mammalian liver, the expression pattern of *postnb* following PH suggests that HSCs could play a role in triggering the early initiation of liver regeneration in male zebrafish (Kumar et al., 2018). For the genes upregulated in the female zebrafish on Day 1, some of the most significantly upregulated genes (e.g. *rnd1b* and *calm1b*) encode inhibitors of cell proliferation, which may contribute to the relatively slow response of hepatocyte proliferation in females compared with males after PH (Orellana et al., 2010; Qin et al., 2018).

In conclusion, the current study confirms that male zebrafish undergo earlier liver cell proliferation and liver mass recovery than female zebrafish during PH-induced liver regeneration. This sex disparity is regulated by sex hormone receptors, including ARs and ERs, and the S100A1-Yap1 signaling cascade. These findings could enhance understanding of how sex affects liver regeneration following PH. Moreover, the current study has identified sex hormone receptors and S100A1 as potential targets for facilitating hepatocyte proliferation during PH-induced liver regeneration, which provides clues for developing novel therapeutic strategies.

## MATERIALS AND METHODS

### Zebrafish husbandry

The maintenance of zebrafish used in the current study follows the Institutional Animal Care and Use Committee guidelines from the Southern University of Science and Technology, China, and the National University of Singapore. Transgenic zebrafish lines including Tg(*fabp10a*:LOXP-EGFP-LOXP-DsRed; TETRE:Cre-ERT2) (gz38Tg) with EGFP-labeled hepatocytes (Li et al., 2019), Tg(*fabp10a*:DsRed; *ela3l*:EGFP) (gz15Tg) with DsRed-labeled hepatocytes and GFP-labeled exocrine pancreas (Korzh et al., 2008), and Tg(*fabp10a*:CFP-NTR) (s931Tg) with liver-specific expression of CFP-linked NTR (Choi et al., 2014) were used in the current study and designated as EGFP^+^, LiPan and NTR^+^, respectively. The genetic backgrounds of the WT zebrafish used in the current study included AB, Tübingen and Singapore indigenous strain, which was initially purchased from a local ornamental fish farm in Singapore and has been maintained in our laboratory for more than 20 years. The exact strains of WT zebrafish used in each experiment are indicated in the text.

### Mutant generation

To generate *s100a1*-KO zebrafish with CRISPR/Cas9-mediated large-fragment deletion, two gRNAs targeting the 5′ UTR and 3′ UTR of the *s100a1* gene were designed with CRISPRscan and synthesized by Genscript Biotech (Nanjing, Jiangsu, China). gRNAs were microinjected together with EnGen Spy Cas9 NLS (M0646, NEB) protein into zebrafish embryos (AB strain) at the one-cell stage to induce deletion of the coding region of *s100a1*. The sequences of the 5′ gRNA and 3′ gRNA are 5′-GGGGGAATTAGGCAAATAGG-3′ and 5′-TATGGGGGTGTGTTGTCATA-3′, respectively. The primers used for PCR validation of *s100a1*-KO are 5′-GTTAAATCACGCAAGTCCAGTCT-3′ (forward) and 5′-AGGGCAAATCAAGTCAGCCAA-3′ (reverse). Gel electrophoresis of PCR products was performed using a standard protocol. Sequencing of PCR products was conducted by Bio Basic Asia Pacific, Singapore.

### Partial hepatectomy and LBR measurement

Adult zebrafish (>12 weeks old) of both sexes were used in the PH experiments. The procedure for performing PH on the zebrafish was derived from a published protocol (Oderberg and Goessling, 2021). In general, after anesthesia with 150 mg l^−1^ buffered tricaine (MS-222), a 2-3 mm incision was made on the zebrafish abdomen with a micro stab knife and precise scissors to expose the liver. Subsequently, the ventral lobe of the liver was removed with tweezers, resulting in ∼30% of the liver being resected. Sham surgery was performed as a control by making an incision on the abdomen without removing the liver tissue. To measure the LBR, euthanized zebrafish were placed on KimWipe papers (Kimberley-Clark, Irving, TX, USA) to remove attached water and then weighed on an analytical balance. Subsequently, zebrafish were dissected, and the livers were collected in pre-weighed Eppendorf tubes separately and weighed again. The liver weight was calculated by subtracting the weight of empty tubes.

### Chemical treatment

Chemicals used in the current study include DHT (S4757, Selleck), fadrozole (HY-14247; MedChemExpress), flutamide (S1908, Selleck), ICI (Fulvestrant) (S1191, Selleck) and VP (HY-B0146, MedChemExpress). Stocks of chemicals were prepared at first by dissolving chemicals in DMSO (D4540, Sigma-Aldrich). Fadrozole, flutamide and ICI were used for treating adult zebrafish from 48 h before the surgery at 100 μg l^−1^, 5 μM and 1 mg l^−1^, respectively, through exposure. The control group for chemical exposure received 0.1% DMSO treatment. For VP and DHT treatments, chemical stocks were diluted to the working concentration in a solvent consisting of 10% DMSO, 45% PBS (P6748, Sigma-Aldrich), 40% PEG300 (HY-Y0873, MedChemExpress) and 5% Tween 80 (HY-Y1891, MedChemExpress). Twelve hours before the surgery, 25 ng VP and/or 200 nM DHT was delivered to adult zebrafish in 5 μl of the solvent by intraperitoneal injection. The control group for VP and DHT treatment was injected with only the solvent. Intraperitoneal injection was conducted following a published protocol (Kinkel et al., 2010) except MS-222 was used for anesthesia.

### RNA extraction and RT-qPCR

Total RNA extraction from tissues and embryos was performed using TRIzol reagent (Invitrogen), followed by reversed transcription using a Transcriptor First Strand cDNA Synthesis Kit (Roche). The synthesized cDNA was used for RT-qPCR with SsoAdvanced Universal Supermixes (Bio-Rad) and GoTaq qPCR Master Mix (Promega) in a CFX96 Touch Real-Time PCR System (Bio-Rad) and ABI 7500 Fast Dx Real-Time PCR Instrument (Thermo Fisher Scientific), respectively. Genes of interest were amplified by 40 cycles using a standard three-step protocol (95°C, 20 s; 65°C, 15 s; 72°C, 30 s). All primers used in RT-qPCR are listed in Table S2.

### RNA-Seq and bioinformatic analysis

Liver tissues collected from WT adult zebrafish after surgery were used for RNA extraction with TRIzol reagent. For RNAs extracted from PH/sham surgery-treated zebrafish, library preparation and Illumina sequencing were conducted by Novogen, Hong Kong. Each sample used for RNA-Seq contained RNAs from three individual zebrafish livers, and each biological group contained two pooled samples. After sequencing, reads were mapped to zebrafish reference genome assembly GRCz11 with TopHat2. The raw counts of sequences mapped to each gene were quantified using StringTie, followed by differential expression analysis using DESeq2 (Love et al., 2014). The criteria of significantly deregulated genes were adjusted *P*-value (padj)≤0.05 and FC≥2. To identify enriched pathways, pre-ranked GSEA was performed using the fgsea package (Korotkevich et al., 2016 preprint). The Hallmark pathway gene sets used for GSEA were downloaded from the Molecular Signature Database (Subramanian et al., 2005). The DEGs were ranked by their values of Wald statistic generated from DESeq2.

### MO knockdown

For knockdown of *s100a1*, the MO targeting the start codon of *s100a1* gene, MO-*s100a1*-ATG (5′-GTGACGACAGCTTGAAAAGATGTT-3′) (Gene Tools, Philomath, OR, USA), was synthesized and applied. The effectiveness of MO-*s100a1*-ATG inhibiting S100A1 expression was validated previously by western blotting (Zhu et al., 2024). Aliquots of MO (50 nM) were injected into zebrafish embryos at the one-cell stage. MTZ treatment was conducted by exposing 3.5 dpf larvae to 10 mM MTZ (S1907, Selleck) in 0.1% DMSO for 24 h. The control larvae were exposed to 0.1% DMSO only.

### Histological analyses

Adult zebrafish liver and gut tissues were harvested together and fixed in 10% formalin and 4% paraformaldehyde in PBS overnight for subsequent paraffin embedding and cryo-embedding, respectively. Paraffin-embedded samples were sectioned at 5 μm with a microtome, and frozen samples were sectioned at 8 μm with a cryostat. All frozen samples were used for IF staining, while paraffin-embedded samples were primarily used for IHC staining. Sections were incubated with primary antibodies overnight at 4°C.. For IHC staining, a DAKO Real Envision Detection System (K500711, Agilent) was used for incubation of horseradish peroxidase (HRP)-conjugated secondary antibody and color development. For IF staining, sections were incubated with fluorescent-dye conjugated secondary antibodies (Alexa Fluor™, Invitrogen) for 2 h at room temperature. The primary antibodies used in IF and IHC include mouse anti-Pcna (sc-56, Santa Cruz Biotechnology) at a dilution of 1:200, mouse anti-E-cadherin (610182, BD Biosciences) at a dilution of 1:200, rabbit anti-Bhmt (ab211119, Abcam) at a dilution of 1:200, rabbit anti-Hnf4α (ab201460, Abcam) at a dilution of 1:2000, and rabbit anti-Yap1 (4912S, Cell Signaling Technology) at a dilution of 1:100. During immunostaining of proteins except for Yap1, tissue sections underwent antigen retrieval by placing in citrate buffer (C9999, Sigma-Aldrich) in 95°C water baths.

### Photography and image analysis

Side views of dissected adult zebrafish were photographed individually with a stereo fluorescence microscope (SZX16, Olympus, Tokyo, Japan). Larvae photography was performed with an Axio Imager 2 light microscope (Carl Zeiss AG, Oberkochen, Germany) after immobilizing larvae in 3% methylcellulose. For IHC staining, imaging was conducted using the Axio Imager 2 light microscope and a NanoZoomer S60 digital slide scanner (Hamamatsu Photonics, Shizuoka, Japan). For imaging IHC staining, at least two high-power fields of view per sample were captured. Tissue sections of IF staining were imaged with an LSM 900 confocal laser scanning microscope (Carl Zeiss AG). For imaging IF staining, at least three high-power fields of view per sample were captured and analyzed. All image analyses were performed with ImageJ (Schneider et al., 2012).

### Statistical analysis

For statistical analysis, Prism 8.0 (GraphPad, San Diego, CA, USA) was applied. Statistical significance was determined by two-tailed unpaired Student’s *t*-test unless stated otherwise.

## Supplementary Material

10.1242/dmm.050900_sup1 Supplementary information

## References

[DMM050900C1] Abu Rmilah, A., Zhou, W., Nelson, E., Lin, L., Amiot, B. and Nyberg, S. L. (2019). Understanding the marvels behind liver regeneration. *Wiley Interdiscip. Rev. Dev. Biol.* 8, e340. 10.1002/wdev.340 30924280 PMC6457252

[DMM050900C2] Batmunkh, B., Choijookhuu, N., Srisowanna, N., Byambatsogt, U., Synn Oo, P., Noor Ali, M., Yamaguchi, Y. and Hishikawa, Y. (2017). Estrogen accelerates cell proliferation through estrogen receptor α during rat liver regeneration after partial Hepatectomy. *Acta Histochem. Cytochem.* 50, 39-48. 10.1267/ahc.17003 28386149 PMC5374102

[DMM050900C3] Birrer, D. L., Linecker, M., López-López, V., Brusadin, R., Navarro-Barrios, Á., Reese, T., Arbabzadah, S., Balci, D., Malago, M., Machado, M. A. et al. (2022). Sex disparities in outcomes following major liver surgery: new powers of estrogen? *Ann. Surg.* 276, 875-881. 10.1097/SLA.000000000000563 35894447

[DMM050900C4] Chaturantabut, S., Shwartz, A., Evason, K. J., Cox, A. G., Labella, K., Schepers, A. G., Yang, S., Acuña, M., Houvras, Y., Mancio-Silva, L. et al. (2019). Estrogen activation of G-protein-coupled estrogen receptor 1 regulates phosphoinositide 3-kinase and mTOR signaling to promote liver growth in zebrafish and proliferation of human hepatocytes. *Gastroenterology* 156, 1788-1804.e13. 10.1053/j.gastro.2019.01.010 30641053 PMC6532055

[DMM050900C5] Chen, F., Huang, D., Shi, H., Gao, C., Wang, Y. and Peng, J. (2020). Capn3 depletion causes Chk1 and Wee1 accumulation and disrupts synchronization of cell cycle reentry during liver regeneration after partial hepatectomy. *Cell Regen.* 9, 8. 10.1186/s13619-020-00049-1 32588143 PMC7306836

[DMM050900C6] Choi, T. Y., Ninov, N., Stainier, D. Y. R. and Shin, D. (2014). Extensive conversion of hepatic biliary epithelial cells to hepatocytes after near total loss of hepatocytes in zebrafish. *Gastroenterology* 146, 776-788. 10.1053/j.gastro.2013.10.019 24148620 PMC3943869

[DMM050900C7] Cinar, B., Al-Mathkour, M. M., Khan, S. A. and Moreno, C. S. (2020). Androgen attenuates the inactivating phospho-Ser-127 modification of yes-associated protein 1 (YAP1) and promotes YAP1 nuclear abundance and activity. *J. Biol. Chem.* 295, 8550-8559. 10.1074/jbc.RA120.013794 32376689 PMC7307196

[DMM050900C8] Dong, J., Ke, M.-Y., Wu, X.-N., Ding, H.-F., Zhang, L.-N., Ma, F., Liu, X.-M., Wang, B., Liu, J.-L., Lu, S.-Y. et al. (2022). SRY is a key mediator of sexual dimorphism in hepatic ischemia/reperfusion injury. *Ann. Surg.* 276, 345-356. 10.1097/SLA.0000000000004422 33086308

[DMM050900C9] Dovey, M., Patton, E., Bowman, T., North, T., Goessling, W., Zhou, Y. and Zon, L. (2009). Topoisomerase IIα is required for embryonic development and liver regeneration in Zebrafish. *Mol. Cell. Biol.* 29, 3746-3753. 10.1128/mcb.01684-08 19380487 PMC2698760

[DMM050900C10] Fouraschen, S. M., de Ruiter, P. E., Kwekkeboom, J., de Bruin, R. W., Kazemier, G., Metselaar, H. J., Tilanus, H. W., van der Laan, L. J. and de Jonge, J. (2013). mTOR signaling in liver regeneration: rapamycin combined with growth factor treatment. *World J Transplant* 3, 36-47. 10.5500/wjt.v3.i3.36 24255881 PMC3832859

[DMM050900C11] Francavilla, A., Panella, C., Polimeno, L., Giangaspero, A., Mazzaferro, V., Pan, C. E., Van Thiel, D. H. and Starzl, T. E. (1990). Hormonal and enzymatic parameters of hepatic regeneration in patients undergoing major liver resections. *Hepatology* 12, 1134-1138. 10.1002/hep.1840120510 2227810 PMC2952466

[DMM050900C12] Gao, C. and Peng, J. (2021). All routes lead to Rome: multifaceted origin of hepatocytes during liver regeneration. *Cell Regen.* 10, 2. 10.1186/s13619-020-00063-3 33403526 PMC7785766

[DMM050900C13] Grijalva, J. L., Huizenga, M., Mueller, K., Rodriguez, S., Brazzo, J., Camargo, F., Sadri-Vakili, G. and Vakili, K. (2014). Dynamic alterations in Hippo signaling pathway and YAP activation during liver regeneration. *Am. J. Physiol. Gastrointest. Liver Physiol.* 307, G196-G204. 10.1152/ajpgi.00077.2014 24875096

[DMM050900C14] Goessling, W., North, T. E., Loewer, S., Lord, A. M., Lee, S., Stoick-Cooper, C. L., Weidinger, G., Puder, M., Daley, G. Q., Moon, R. T. et al. (2009). Genetic interaction of PGE2 and Wnt signaling regulates developmental specification of stem cells and regeneration. *Cell* 136, 1136-1147. 10.1016/j.cell.2009.01.015 19303855 PMC2692708

[DMM050900C15] Haga, S., Ogawa, W., Inoue, H., Terui, K., Ogino, T., Igarashi, R., Takeda, K., Akira, S., Enosawa, S., Furukawa, H. et al. (2005). Compensatory recovery of liver mass by Akt-mediated hepatocellular hypertrophy in liver-specific STAT3-deficient mice. *J. Hepatol.* 43, 799-807. 10.1016/j.jhep.2005.03.027 16083985

[DMM050900C16] Hansen, C. G., Moroishi, T. and Guan, K. L. (2015). YAP and TAZ: a nexus for Hippo signaling and beyond. *Trends Cell Biol.* 25, 499-513. 10.1016/j.tcb.2015.05.002 26045258 PMC4554827

[DMM050900C17] Jin, H. J., Kim, J. and Yu, J. (2013). Androgen receptor genomic regulation. *Transl. Androl. Urol.* 2, 157-177. 10.3978/j.issn.2223-4683.2013.09.01 25237629 PMC4165347

[DMM050900C18] Kan, N., Junghans, D. and Belmonte, J. (2009). Compensatory growth mechanisms regulated by BMP and FGF signaling mediate liver regeneration in zebrafish after partial hepatectomy. *FASEB J.* 23, 3516-3525. 10.1096/fj.09-131730 19546304 PMC2747679

[DMM050900C19] Kao, T., Chen, Y., Kuan, Y., Chang, W., Ho, Y., Yeh, S., Jeng, L.-B. and Ma, W.-L. (2017). Estrogen–estrogen receptor α signaling facilitates bilirubin metabolism in regenerating liver through regulating cytochrome P450 2A6 expression. *Cell Transplant.* 26, 1822-1829. 10.1177/0963689717738258 29338386 PMC5784527

[DMM050900C20] Kinkel, M. D., Eames, S. C., Philipson, L. H. and Prince, V. E. (2010). Intraperitoneal injection into adult zebrafish. *J. Vis. Exp.* 42, 2126. 10.3791/2126 PMC3278329 20834219

[DMM050900C21] Korotkevich, G., Sukhov, V., Budin, N., Shpak, B., Artyomov, M. N. and Sergushichev, A. (2016). Fast gene set enrichment analysis. *bioRxiv*. 10.1101/060012

[DMM050900C22] Korzh, S., Pan, X., Garcia-Lecea, M., Winata, C. L., Pan, X., Wohland, T., Korzh, V. and Gong, Z. (2008). Requirement of vasculogenesis and blood circulation in late stages of liver growth in zebrafish. *BMC Dev. Biol.* 8, 84. 10.1186/1471-213X-8-84 18796162 PMC2564926

[DMM050900C23] Kowalczyk, W., Romanelli, L., Atkins, M., Hillen, H., Bravo González-Blas, C., Jacobs, J., Xie, J., Soheily, S., Verboven, E., Moya, I. M. et al. (2022). Hippo signaling instructs ectopic but not normal organ growth. *Science* 378, eabg3679. 10.1126/science.abg3679 36395225

[DMM050900C24] Kumar, P., Smith, T., Raeman, R., Chopyk, D. M., Brink, H., Liu, Y., Sulchek, T. and Anania, F. A. (2018). Periostin promotes liver fibrogenesis by activating lysyl oxidase in hepatic stellate cells. *J. Biol. Chem.* 293, 12781-12792. 10.1074/jbc.RA117.001601 29941453 PMC6102155

[DMM050900C25] Kuser-Abali, G., Alptekin, A., Lewis, M., Garraway, I. P. and Cinar, B. (2015). YAP1 and AR interactions contribute to the switch from androgen-dependent to castration-resistant growth in prostate cancer. *Nat. Commun.* 6, 8126. 10.1038/ncomms9126 28230103 PMC5327734

[DMM050900C26] Labgaa, I., Taffé, P., Martin, D., Clerc, D., Schwartz, M., Kokudo, N., Denys, A., Halkic, N., Demartines, N. and Melloul, E. (2020). Comparison of partial hepatectomy and transarterial chemoembolization in intermediate-stage hepatocellular carcinoma: a systematic review and meta-analysis. *Liver Cancer* 9, 138-147. 10.1159/000505093 32399428 PMC7206581

[DMM050900C27] Li, Y., Agrawal, I. and Gong, Z. (2019). Reversion of tumor hepatocytes to normal hepatocytes during liver tumor regression in an oncogene-expressing transgenic zebrafish model. *Dis. Model. Mech.* 12, dmm039578. 10.1242/dmm.039578 31515263 PMC6826027

[DMM050900C28] Li, X., Nie, S., Lv, Z., Ma, L., Song, Y., Hu, Z., Hu, X., Liu, Z., Zhou, G., Dai, Z. et al. (2021). Overexpression of Annexin A2 promotes proliferation by forming a Glypican 1/c-Myc positive feedback loop: prognostic significance in human glioma. *Cell Death Dis.* 12, 261. 10.1038/s41419-021-03547-5 33712571 PMC7954792

[DMM050900C29] Liberzon, A., Birger, C., Thorvaldsdóttir, H., Ghandi, M., Mesirov, J. and Tamayo, P. (2015). The molecular signatures database hallmark gene set collection. *Cell Syst.* 1, 417-425. 10.1016/j.cels.2015.12.004 26771021 PMC4707969

[DMM050900C30] Liu, C., Cheng, X., Chen, J., Wang, Y., Wu, X., Tian, R., Liu, B., Ding, X., Sun, Q. and Gong, W. (2019). Suppression of YAP/TAZ/Notch1-NICD axis by bromodomain and extraterminal protein inhibition impairs liver regeneration. *Theranostics* 9, 3840-3852. 10.7150/thno.33370 31281517 PMC6587347

[DMM050900C31] Love, M. I., Huber, W. and Anders, S. (2014). Moderated estimation of fold change and dispersion for RNA-seq data with DESeq2. *Genome Biol.* 15, 550. 10.1186/s13059-014-0550-8 25516281 PMC4302049

[DMM050900C32] Lu, L., Finegold, M. J. and Johnson, R. L. (2018). Hippo pathway coactivators yap and Taz are required to coordinate mammalian liver regeneration. *Exp. Mol. Med.* 50, e423-e423. 10.1038/emm.2017.205 29303509 PMC5992983

[DMM050900C33] Lu, H., Yin, M., Wang, L., Cheng, J., Cheng, W., An, H. and Zhang, T. (2020). FGF13 interaction with SHCBP1 activates AKT-GSK3α/β signaling and promotes the proliferation of A549 cells. *Cancer Biol. Ther.* 21, 1014-1024. 10.1080/15384047.2020.1824512 33064958 PMC7678946

[DMM050900C34] Luther, N. (2010). Insulin-like growth factor binding protein-3 is required for the regulation of Rat Oval cell proliferation and differentiation in the 2AAF/PHX model. *Hepat. Med.* 2010, 13-32. 10.2147/HMER.S7660 21852899 PMC3156464

[DMM050900C35] Marongiu, F., Marongiu, M., Contini, A., Serra, M., Cadoni, E., Murgia, R. and Laconi, E. (2017). Hyperplasiavshypertrophy in tissue regeneration after extensive liver resection. *World J. Gastroenterol.* 23, 1764. 10.1016/wjg.v23.i10.1764 28348481 PMC5352916

[DMM050900C36] Michalopoulos, G. K. (2007). Liver regeneration. *J. Cell. Physiol.* 213, 286-300. 10.1002/jcp.21172 17559071 PMC2701258

[DMM050900C37] Miyaoka, Y., Ebato, K., Kato, H., Arakawa, S., Shimizu, S. and Miyajima, A. (2012). Hypertrophy and unconventional cell division of hepatocytes underlie liver regeneration. *Curr. Biol.* 22, 1166-1175. 10.1016/j.cub.2012.05.016 22658593

[DMM050900C38] Nishi, K., Fu, W. and Kiyama, R. (2022). Novel estrogen-responsive genes (ERGs) for the evaluation of estrogenic activity. *PLoS One* 17, e0273164. 10.1371/journal.pone.0273164 35976950 PMC9385026

[DMM050900C39] Oderberg, I. M. and Goessling, W. (2021). Partial hepatectomy in adult zebrafish. *J. Vis. Exp.* 170, e62349. 10.3791/62349 PMC8139180 33871461

[DMM050900C40] Qin, C.-D., Ma, D.-N., Zhang, S.-Z., Zhang, N., Ren, Z.-G., Zhu, X.-D., Jia, Q.-A., Chai, Z.-T., Wang, C.-H., Sun, H.-C. et al. (2018). The Rho GTPase Rnd1 inhibits epithelial-mesenchymal transition in hepatocellular carcinoma and is a favorable anti-metastasis target. *Cell Death Dis.* 9, 486. 10.1038/s41419-018-0517-x 29706627 PMC5924761

[DMM050900C41] Orellana, D., Liu, X., Wang, G., Jin, J., Iakova, P. and Timchenko, N. (2010). Calmodulin controls liver proliferation via interactions with C/EBPβ-LAP and C/EBPβ-LIP. *J. Biol. Chem.* 285, 23444-23456. 10.1074/jbc.m110.129825 20498378 PMC2906335

[DMM050900C42] Sadler, K., Krahn, K., Gaur, N. and Ukomadu, C. (2007). Liver growth in the embryo and during liver regeneration in zebrafish requires the cell cycle regulator, uhrf1. *Proc. Natl. Acad. Sci. USA* 104, 1570-1575. 10.1073/pnas.0610774104 17242348 PMC1785278

[DMM050900C43] Saito, T., Ikeda, T., Nakamura, K., Chung, U. I. and Kawaguchi, H. (2007). S100A1 and S100B, transcriptional targets of SOX trio, inhibit terminal differentiation of chondrocytes. *EMBO Rep.* 8, 504-509. 10.1038/sj.embor.7400934 17396138 PMC1866207

[DMM050900C44] Schneider, C. A., Rasband, W. S. and Eliceiri, K. W. (2012). NIH image to ImageJ: 25 years of image analysis. *Nat. Methods* 9, 671-675. 10.1038/nmeth.2089 22930834 PMC5554542

[DMM050900C45] Scott, C. E. and Kekenes-Huskey, P. M. (2016). Molecular basis of S100A1 activation at saturating and subsaturating calcium concentrations. *Biophys. J.* 110, 1052-1063. 10.1016/j.bpj.2015.12.040 26958883 PMC4788715

[DMM050900C46] Subramanian, A., Tamayo, P., Mootha, V. K., Mukherjee, S., Ebert, B. L., Gillette, M. A., Paulovich, A., Pomeroy, S. L., Golub, T. R., Lander, E. S. et al. (2005). Gene set enrichment analysis: a knowledge-based approach for interpreting genome-wide expression profiles. *Proc. Natl. Acad. Sci. U.S.A.* 102, 15545-15550. 10.1073/pnas.0506580102 16199517 PMC1239896

[DMM050900C47] Tsukamoto, I. and Kojo, S. (1990). The sex difference in the regulation of liver regeneration after partial hepatectomy in the rat. *Biochim. Biophys. Acta – Gen. Subj.* 1033, 287-290. 10.1016/0304-4165(90)90135-j 2317504

[DMM050900C48] Uebi, T., Umeda, M. and Imai, T. (2015). Estrogen induces estrogen receptor alpha expression and hepatocyte proliferation in the livers of male mice. *Genes Cells* 20, 217-223. 10.1111/gtc.12214 25495062

[DMM050900C49] Wang, Y., Ye, F., Ke, Q., Wu, Q., Yang, R. and Bu, H. (2013). Gender-dependent histone deacetylases injury may contribute to differences in liver recovery rates of male and female mice. *Transplant. Proc.* 45, 463-473. 10.1016/j.transproceed.2012.06.063 23498780

[DMM050900C51] Wang, Y., Huang, X. Z., He, L., Pu, W., Li, Y., Liu, Q., Li, Y., Zhang, L., Yu, W., Zhao, H. et al. (2017). Genetic tracing of hepatocytes in liver homeostasis, injury, and regeneration. *J. Biol. Chem.* 292, 8594-8604. 10.1074/jbc.m117.782029 28377509 PMC5448089

[DMM050900C52] Wang, G., Li, H.-N., Cui, X.-Q., Xu, T., Dong, M.-L., Li, S.-Y. and Li, X.-R. (2021). S100A1 is a potential biomarker for papillary thyroid carcinoma diagnosis and prognosis. *J. Cancer* 12, 5760-5771. 10.7150/jca.51855 34475990 PMC8408122

[DMM050900C53] Wu, T., Huang, J., Wu, S., Huang, Z., Chen, X., Liu, Y., Cui, D., Song, G., Luo, Q., Liu, F. et al. (2018). Deficiency of periostin impairs liver regeneration in mice after partial hepatectomy. *Matrix Biol.* 66, 81-92. 10.1016/j.matbio.2017.09.004 28965986

[DMM050900C59] Yamaguchi, M., Yu, L., El-Assal, O. N., Satoh, T., Dhar, D. K., Yamanoi, A. and Nagasue, N. (2001). Androgen metabolism in regenerating liver of male rats: evidence for active uptake and utilization of testosterone. *Hepatol. Res.* 20, 114-127. 10.1016/s1386-6346(00)00125-x 11282490

[DMM050900C54] Zhou, Y., Zhang, L., Ji, H., Lu, X., Xia, J., Li, L., Chen, F., Bu, H. and Shi, Y. (2016). MiR-17∼92 ablation impairs liver regeneration in an estrogen-dependent manner. *J. Cell. Mol. Med.* 20, 939-948. 10.1111/jcmm.12782 26781774 PMC4831359

[DMM050900C55] Zhu, Z., Chen, J., Xiong, J. and Peng, J. (2014). Haploinsufficiency of Def activates p53-dependent TGFβ signalling and causes scar formation after partial hepatectomy. *PloS One* 9, e96576. 10.1371/journal.pone.0096576 24801718 PMC4011785

[DMM050900C56] Zhu, M., Li, Y., Liu, D. and Gong, Z. (2024). Partial hepatectomy promotes the development of KRASG12V-Induced hepatocellular carcinoma in zebrafish. *Cancers* 16, 1793. 10.3390/cancers16101793 38791872 PMC11119731

