# Supplementary material for: Sex hormone receptors, calcium-binding protein and Yap1 signaling regulate sex-dependent liver cell proliferation following partial hepatectomy
Source: Dis Model Mech. 2024 Oct 30;17(10):dmm050900. doi: 10.1242/dmm.050900 (PMC11556313; doi:10.1242/dmm.050900)
Supplement: Supplementary information [file dmm-17-050900-s1.pdf]

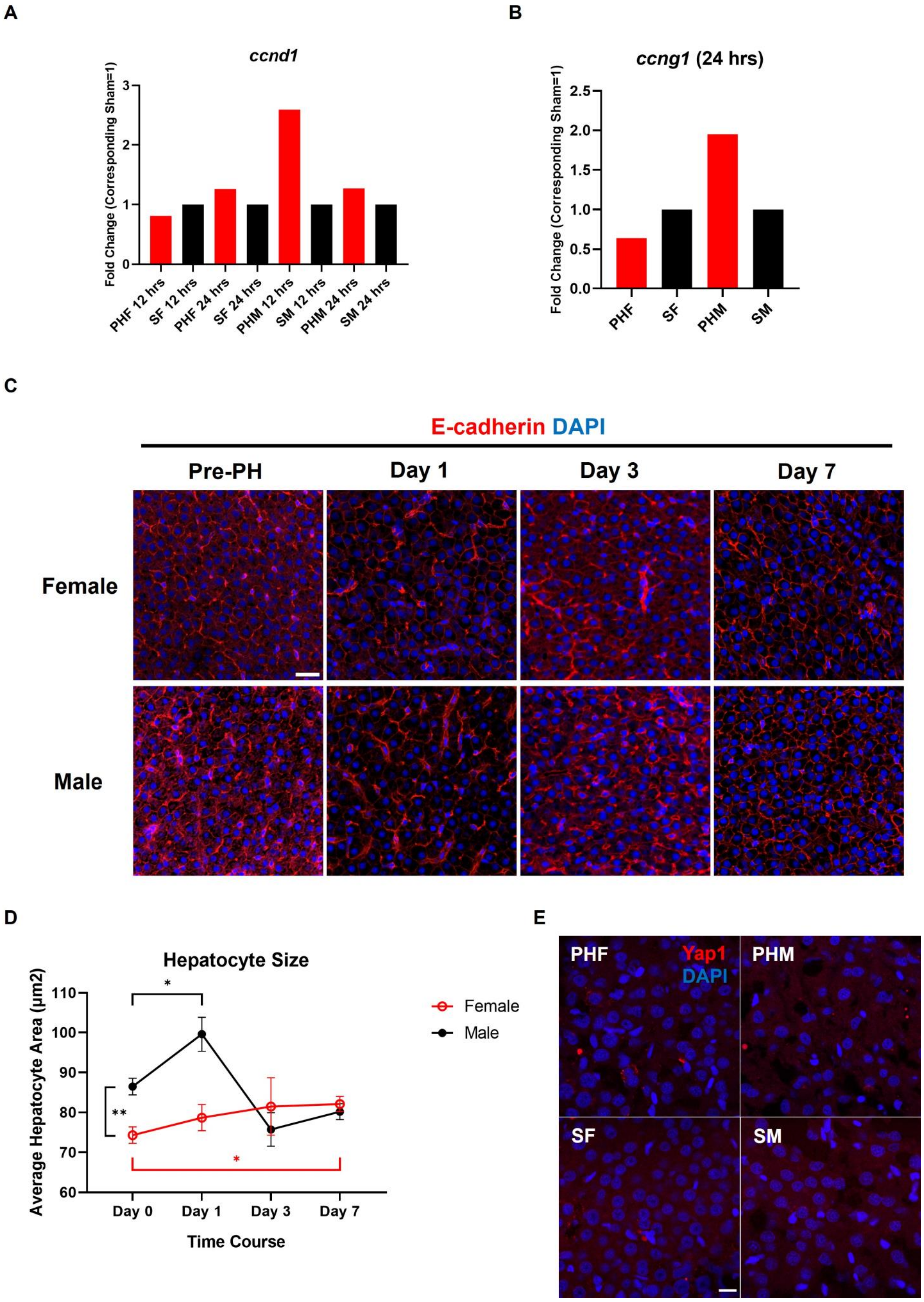

Disease Models & Mechanisms • Supplementary information

Fig. S1. Sex disparity in the expression of cyclins and hepatocyte hypertrophy during PH-induced zebrafish liver regeneration. (A) Expression of *ccnd1* in zebrafish livers of both sexes following PH or sham surgery as determined by RT-qPCR. (B) Expression of *ccng1* in zebrafish livers of both sexes at 24 hours post-PH as determined by RT-qPCR. Each biological group used for qPCR in (A-B) contained one cDNA sample pooled from 3 zebrafish livers. (C) Immunofluorescence (IF) staining of E-cadherin in zebrafish livers of both sexes before and after PH. (D) Measurement of the average hepatocyte size during PH-induced liver regeneration based on (C) (n=3 for Day 7, n=4 for Day 0, n=5 for the remaining groups). (E) IF staining of Yap1 in the zebrafish liver of both sexes at 72 hours post-PH/sham surgery. Scale Bars=20 μm and 10 μm for (C) and (E), respectively. *$P \leq 0.05$, **$P \leq 0.01$ (two-tailed unpaired Student's *t*-test).

Disease Models & Mechanisms • Supplementary information

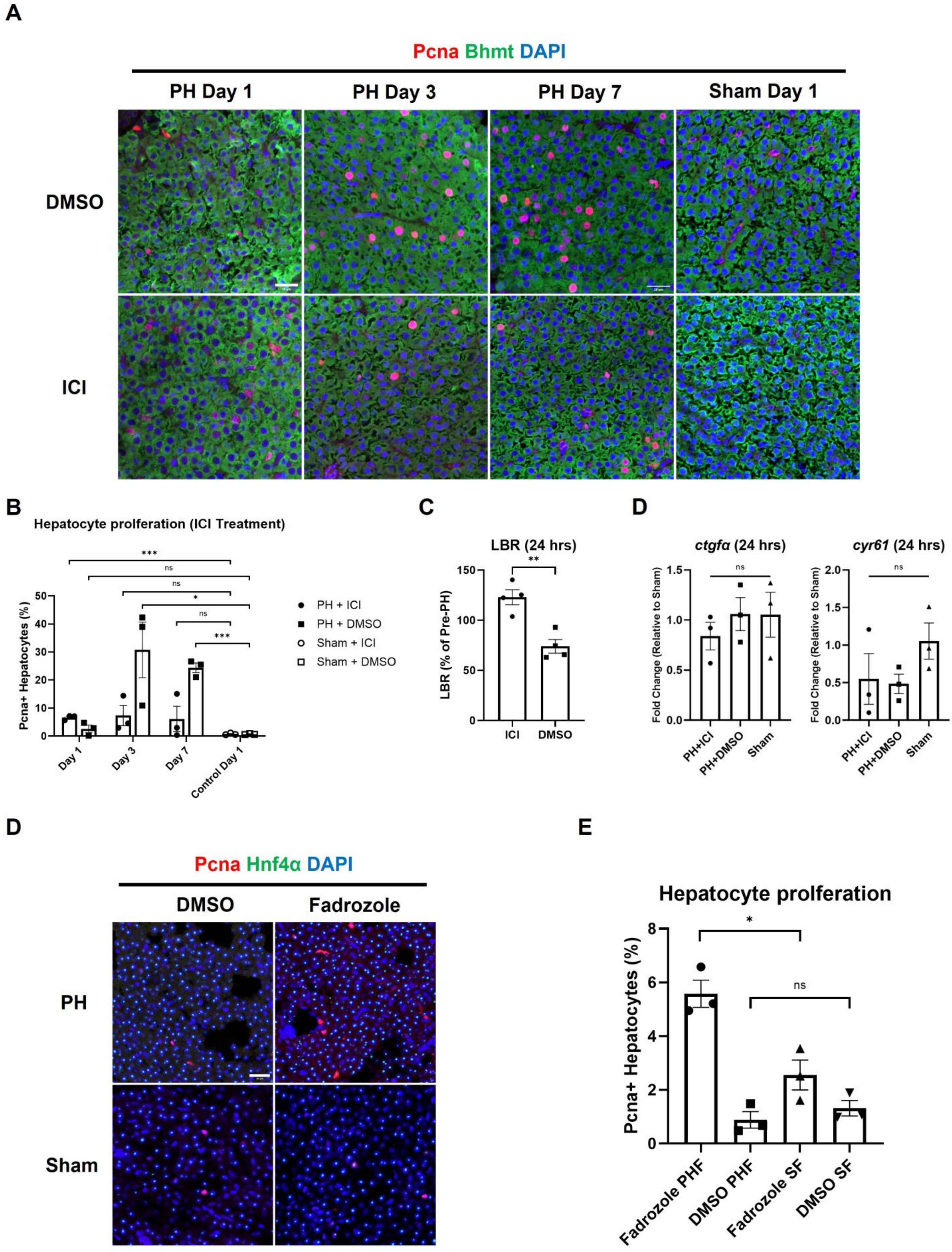

Disease Models & Mechanisms • Supplementary information

Fig. S2. Inhibiting estrogen receptor (ER) activity facilitate the initiation of liver regeneration in female zebrafish following PH. (A) IF staining of Pcna and Bhmt in the dimethyl sulfoxide (DMSO)/ICI 182, 780 (ICI)-treated female zebrafish livers following PH and sham surgery. (B) Quantification of Pcna and Bhmt-double positive proliferating hepatocytes based on (A) (n=3). (C) Liver-to-body ratio (LBR) of DMSO/ICI-treated female zebrafish at 24 hours post-PH. (D) Expression of *ctgfa and cyr61* in the ICI/DMSO-treated female livers at 24 hours post-PH as determined by RT-qPCR (n=3). (E) IF staining of Pcna and Hnf4α in the DMSO/fadrozole-treated female zebrafish livers at 24 hours post-PH/sham surgery. (F) Quantification of Pcna and Hnf4α-double positive proliferating hepatocytes based on (E) (n=3). Scale Bar=20 µm. ns, not significant ($P>0.05$), *$P\leq0.05$, **$P\leq0.01$, ***$P\leq0.001$ (two-tailed unpaired Student`s *t*-test).

Disease Models & Mechanisms • Supplementary information

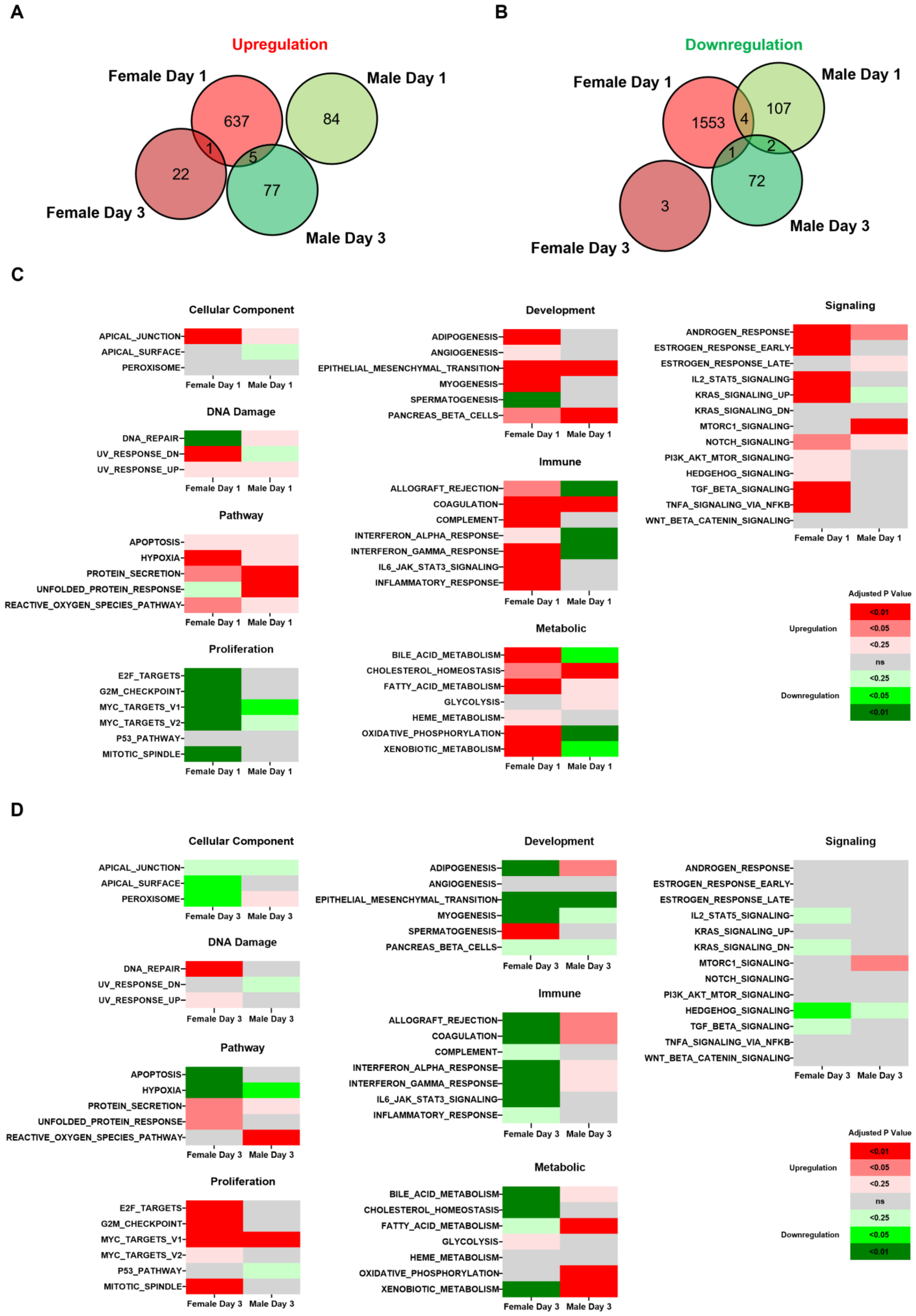

Disease Models & Mechanisms • Supplementary information

Fig. S3. Sex disparity in the transcriptomic regulation during PH-induced zebrafish liver regeneration. (A-B) Venn diagrams showing the overlaps of up/downregulated genes among different groups across the examined time points based on the results of RNA-sequencing (RNA-Seq). (C-D) Comparison of gene set enrichment analysis (GSEA) of Hallmark gene sets between the female liver and the male liver on (C) Day 1 and (D) Day 3 following PH (two-tailed unpaired Student's *t*-test).

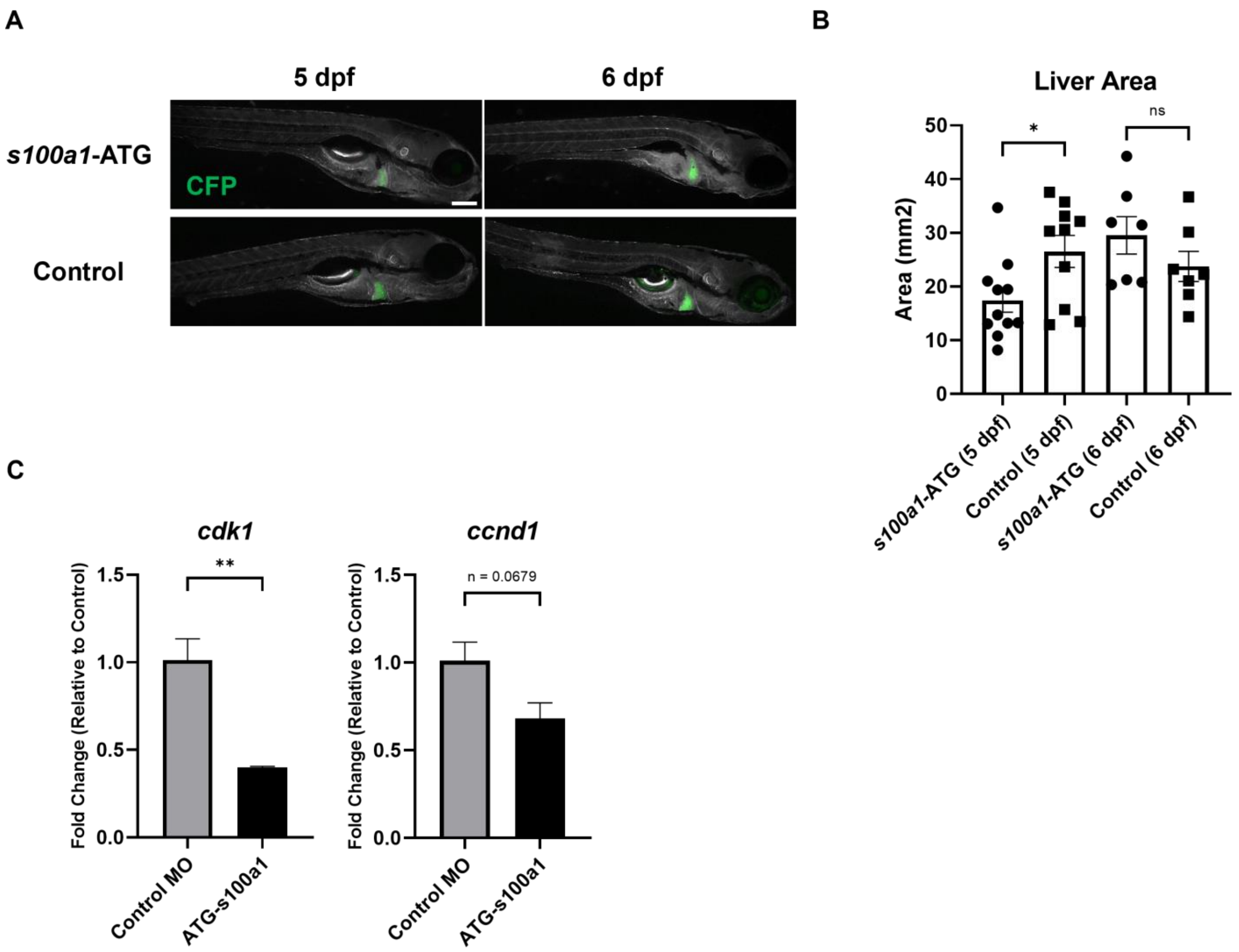

Fig. S4. Effects of *s100a1* knockdown on liver expansion during zebrafish embryonic development. (A) Fluorescence images of morpholino (MO)-injected NTR+ larvae at 5 days post fertilization (dpf) and 6 dpf. (B) Measurement of the 2D area of CFP+ livers in MO-injected NTR+ larvae based on (A). Each symbol represents one larva. (C) Expression of *cdk1 and ccnd1* in the MO-injected NTR+ larvae at 4.5 dpf as determined by RT-qPCR (n=3). Each sample used for quantitative PCR contained cDNA from 20 larvae. Scale Bar=200 µm. ns, not significant (P>0.05), *P≤0.05, **P≤0.01 (two-tailed unpaired Student`s t-test).

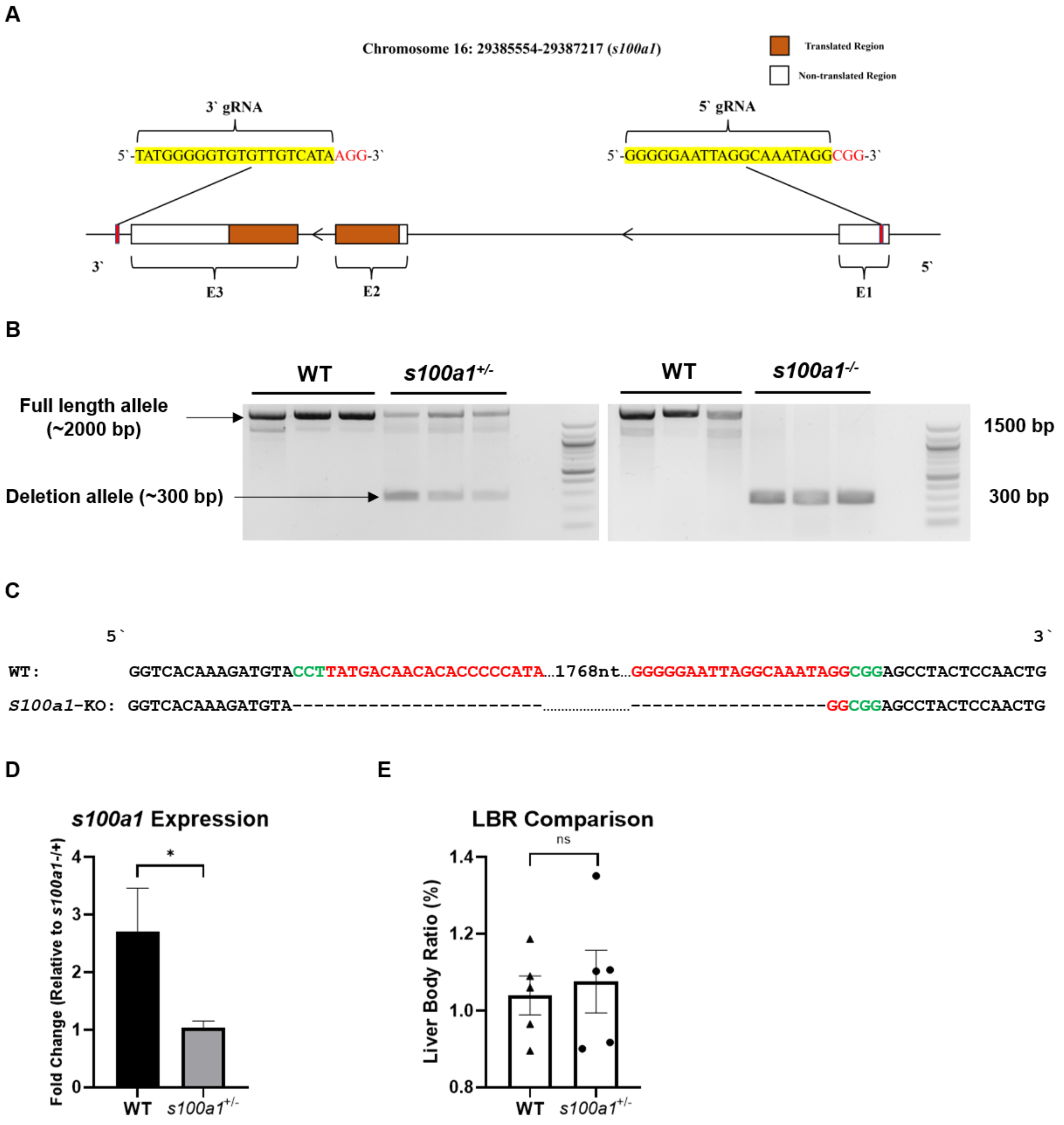

Fig. S5. Confirmation of CRISPR/Cas9-mediated deletion of *s100a1* gene in zebrafish. (A) Illustration of CRISPR/Cas9-mediated large fragment deletion of *s100a1*. Red lines indicate the designed cut sites. Red letters indicate the protospacer adjacent motif (PAM) sequences. (B) PCR examination of *s100a1* genes in the genomes of WT male livers and *s100a1*-knockout (*s100a1*-KO) male livers. (C) Sequencing analysis of *s100a1*-KO males compared with WT males. The presented sequence of *s100a1*-KO zebrafish is identical among three sampled genomes. Red: guide RNA (gRNA) targeting sequence; Green: PAM sequence; Hyphen: deleted region. (D) Expression of *s100a1* in the livers of WT males and *s100a1*$^{+/-}$ males determined by RT-qPCR (n=3). (E) Comparison of LBR in WT males and *s100a1*$^{+/-}$ males. Each symbol represents one zebrafish. ns, not significant (P>0.05), *P≤0.05 (two-tailed unpaired Student`s t-test).

Disease Models & Mechanisms • Supplementary information

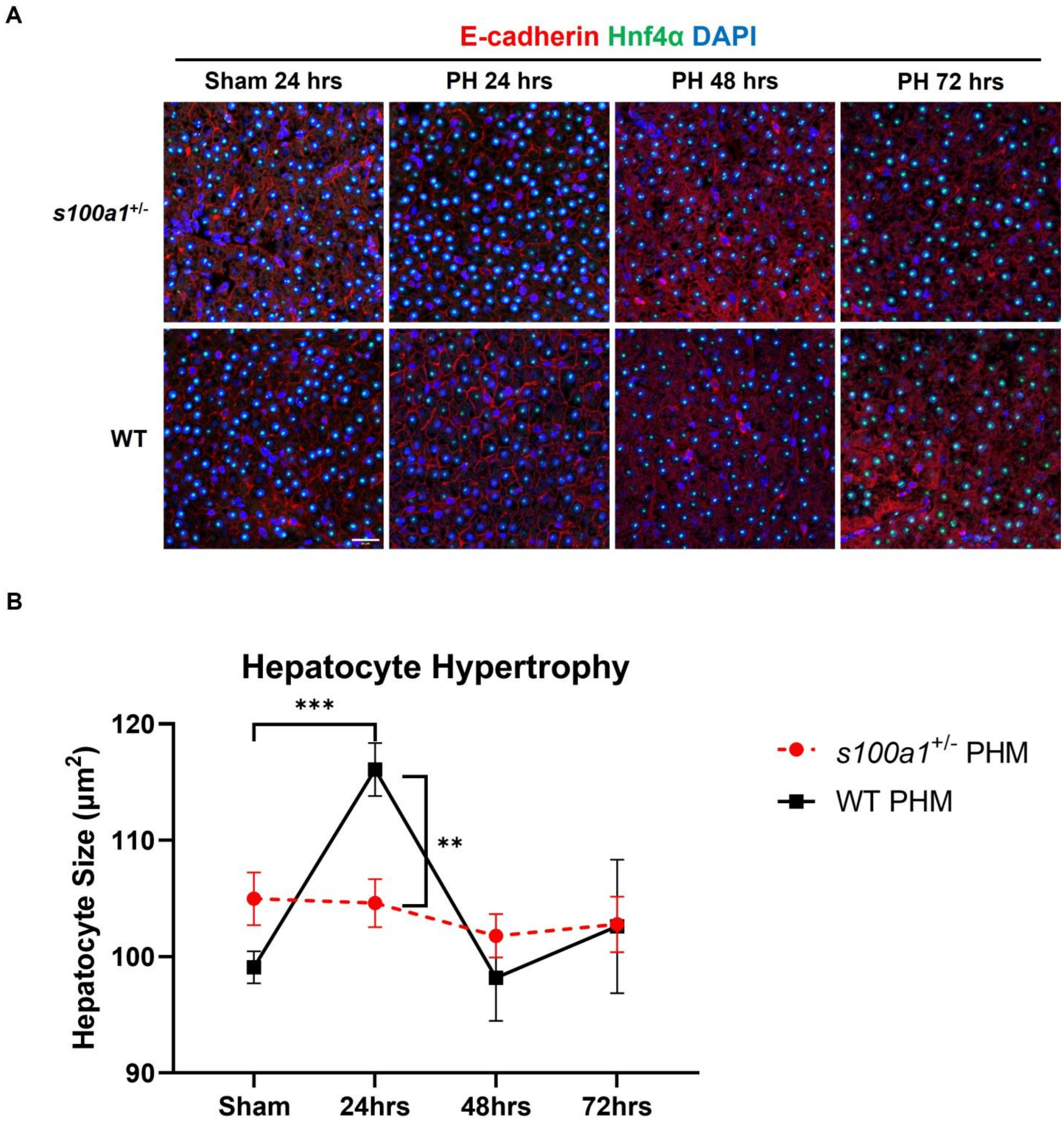

Fig. S6. Effects of *s100a1* haploinsufficiency on male zebrafish hepatocyte hypertrophy following PH. (A) IF staining of E-cadherin and Hnf4α in the *s100a1*$^{+/-}$ and WT zebrafish livers of two sexes at 24 hours, 48 hours, and 72 hours post-PH. (B) Measurement of the average hepatocyte size during PH-induced liver regeneration based on (A) (n=4). Scale Bar=20 μm. ***P*≤0.01, ****P*≤0.001 (two-tailed unpaired Student\`s *t*-test).

Table S1. Top 20 genes upregulated only in male/female livers on Day 1 after partial hepatectomy (PH) ordered by significance

**Genes upregulated only in male livers**	**Male PH vs sham**		**Female PH vs sham**	
**Gene ID**	**log2FoldChange**	**padj**	**log2FoldChange**	**padj**
fgf13b	5.794282	9.44E-15	-0.25067	0.894107
tlr18	4.270187	6.98E-09	-0.54739	0.812264
sst1.2	3.284441	0.000226	0.740665	0.868036
BX511067.1	24.48908	0.000226	-8.03369	0.107028
si:ch211-270n8.1	2.023279	0.000298	-0.14332	0.953256
cxcl8b.3	2.276303	0.001025	-0.30642	0.837407
fkbp2	1.939463	0.001187	0.940029	0.153433
asns	2.458471	0.001529	0.400327	0.587405
BX322792.1	9.088049	0.00182	-0.14853	0.959576
mvda	3.679515	0.00182	0.043186	0.988421
BX571955.2	9.060113	0.001868	-0.25017	0.970659
ntd5	2.724144	0.001891	0.807779	0.741499
s100a1	6.020058	0.001989	-4.66793	4.18E-23
dbi	1.962446	0.00271	0.587476	0.556898
hp	3.724809	0.003252	0.187578	0.97642
anxa2a	1.570429	0.004061	0.305516	0.822361
crp2	2.253436	0.004189	0.29871	0.892893
ppp1r15b	4.322528	0.004861	-0.10564	0.96812
abcc6b.2	1.881603	0.005368	-0.01585	0.99239
sox4b	1.820116	0.00579	-0.0287	0.989585
**Genes upregulated only in female livers**	**Female PH vs sham**		**Male PH vs sham**	
**Gene ID**	**log2FoldChange**	**padj**	**log2FoldChange**	**padj**
rnd1b	4.165381	3.15E-29	-0.02579	0.999902
si:ch73-281i18.7	3.324381	8.29E-10	-1.50476	0.8933
si:ch73-281i18.3	3.513456	9.09E-10	-0.56509	0.999902
socs3b	2.490229	1.24E-09	0.613077	0.894248
calm1b	3.152896	2.19E-09	0.779694	0.798316
zgc:65894	4.653589	1.06E-08	0.183819	0.999902
comtb	2.371968	1.52E-08	0.257692	0.999902
tnfaip2b	6.065512	6.62E-08	0.506349	0.999902
abca1b	2.995347	7.67E-08	-0.09204	0.999902
CR847953.1	3.591262	1.66E-07	0.896738	0.959637
epd	27.95279	2.31E-07	0.866018	0.999902
tnip2	2.362706	3.16E-07	0.20435	0.999902
mpz	27.18037	5.82E-07	0.176665	0.999902
smad1	2.384413	1.02E-06	0.444353	0.999902
prom1b	10.00155	1.12E-06	-4.36455	NA
igflr1	3.113122	1.13E-06	-0.51888	0.999902
hspb6	5.262015	1.13E-06	0.908514	0.999902
mob2a	2.256422	2.35E-06	0.173264	0.999902
cxcr3.3	2.344395	3.32E-06	0.806852	0.867468
il11a	5.28263	6.15E-06	0.269845	0.999902

Table S2. List of primers used in real-time quantitative PCR (RT-qPCR)

**Genes**	**Forward**	**Reverse**
amotl2b	TCCCAGCACAACAGACTTCC	CCGTTTGTCCCTCTAGCTCC
ankrd1b	TGGGAATCCTGCAGGTCGAA	TTGTCAGTATCAAGGCCGCT
ccnd1	TTCCTTGCCAAACTGCCTAT	GGTGAGGTTCTGGGATGAGA
ccng1	GCTCAACTGGAAGGTCAAGG	CAGGGCCAGAAGAGACAAG
cdk1	CTCTGGGGACCCCTAACAAT	CGGATGTGTCATTGCTTGTC
ctgfa	CTACGGCTCCCCAAGTAACC	TCCACTGCGGTACACCATTC
cyp19a1b	TGCACAAACTCAAAAACAAACACAA	CCCAGTATTTAGTTGTTCTTGCCA
cyp24a1	TCCTAATGCTCAACAGTCAG	TTATAGTCACGCAGAATCCA
cyr61	ACAAGCTGCAACCTACCACT	AGAGTATTCATTCTACTCACACTCA
hsd11b2	TTATCAACACACTTCGTCAC	TCTCCAGCAGATATTCTTCC
pcna	GGCAACATCAAGCTCTCACA	TGCAATTTTGTCCTCAACCA
rps18	ATACAGCCAGGTCCTTGCTAATG	GTGACGGAGACCACGGTGAG
s100a1	CCTAATTCCCCCACACAGGT	TTATCCACCAGACGAGGAGCG
sox5	GACTCCTGCAACTCAGACCC	CGTCGCCATGACTACCTCTC
sox9a	CTCAGCCCGAGCCATTACAA	CCTGGTGGCTGTCGGAATAG
sox9b	ACAAATACCAGCCCAGACGG	CTCTGAACAGCGCATTGGTG
sult2st3	CCTTGAAGACCCCGGAACAT	GCACAAGACTCCACGAAGGT
tmprss2	GATGGGGAGCGCTGTTTAGT	GTGTCGGTGATCAGTCCGTT
yap1	ACGGGTGGGAACAAGCTATT	CCTTGCTTTACTGGGGCACT
